# T cell activation is insufficient to drive SIV disease progression

**DOI:** 10.1172/jci.insight.161111

**Published:** 2023-07-24

**Authors:** Cristian Apetrei, Thaidra Gaufin, Egidio Brocca-Cofano, Ranjit Sivanandham, Paola Sette, Tianyu He, Sindhuja Sivanandham, Natalie Martinez Sosa, Kathryn J. Martin, Kevin D. Raehtz, Adam J. Kleinman, Audrey Valentine, Noah Krampe, Rajeev Gautam, Andrew A. Lackner, Alan L. Landay, Ruy M. Ribeiro, Ivona Pandrea

**Affiliations:** 1 Division of Infectious Diseases, Department of Medicine, and; 2 Department of Infectious Diseases and Microbiology, Graduate School of Public Health, University of Pittsburgh, Pittsburgh, Pennsylvania, USA.; 3 Tulane National Primate Research Center, Tulane University, Covington, Louisiana, USA.; 4 Department of Pathology, School of Medicine, University of Pittsburgh, Pittsburgh, Pennsylvania, USA.; 5 Department of Internal Medicine, Rush University Medical Center, Chicago, Illinois, USA.; 6 Los Alamos National Laboratory, Los Alamos, New Mexico, USA.

**Keywords:** AIDS/HIV, Infectious disease, Adaptive immunity, Innate immunity, T cells

## Abstract

Resolution of T cell activation and inflammation is a key determinant of the lack of SIV disease progression in African green monkeys (AGMs). Although frequently considered together, T cell activation occurs in response to viral stimulation of acquired immunity, while inflammation reflects innate immune responses to mucosal injury. We dissociated T cell activation from inflammation through regulatory T cell (Treg) depletion with Ontak (interleukin-2 coupled with diphtheria toxin) during early SIV infection of AGMs. This intervention abolished control of T cell immune activation beyond the transition from acute to chronic infection. Ontak had no effect on gut barrier integrity, microbial translocation, inflammation, and hypercoagulation, despite increasing T cell activation. Ontak administration increased macrophage counts yet decreased their activation. Persistent T cell activation influenced SIV pathogenesis, shifting the ramp-up in viral replication to earlier time points, prolonging the high levels of replication, and delaying CD4^+^ T cell restoration yet without any clinical or biological sign of disease progression in Treg-depleted AGMs. Thus, by inducing T cell activation without damaging mucosal barrier integrity, we showed that systemic T cell activation per se is not sufficient to drive disease progression, which suggests that control of systemic inflammation (likely through maintenance of gut integrity) is the key determinant of lack of disease progression in natural hosts of SIVs.

## Introduction

Chronic T cell immune activation and inflammation are key features of HIV-1 infection in humans and SIV infection of rhesus macaques (RMs) (1–3). T cell activation and inflammation occur early after HIV/SIV infections, increase throughout infection and with disease progression to AIDS, and persist even in individuals receiving antiretroviral therapy (ART) (4, 5). Chronic T cell activation and inflammation are the best independent predictors of progression to AIDS, better than plasma viral loads (pVLs) or CD4^+^ T cell counts, being closely associated with non-AIDS-associated comorbidities and mortality in both HIV and SIV infections, even on ART (6–10).

There are multiple determinants of HIV/SIV-associated chronic T cell activation and inflammation: (i) persistently increased interferon responses (11); (ii) damages to the mucosal barrier, leading to microbial translocation (12–15); (iii) preferential depletion of the Th17 cells involved in preservation of mucosal integrity (16, 17); (iv) coinfections with other pathogens (i.e., cytomegalovirus) (18); and (v) ART toxicity and other drug-related risk factors (6). Of these, persistent damage to the gastrointestinal (GI) tract barrier, and the subsequent microbial translocation, are key pathogenic determinants of HIV/SIV disease progression (13, 19).

Although T cell immune activation and inflammation are usually considered synonymous, they involve different mechanisms: activation of adaptive immunity (T cell immune activation) or of innate immunity (inflammation). Distinguishing these processes may lead to targeted approaches to reverse the deleterious consequences of HIV/SIV infection.

Innate cell activation triggers HIV/SIV-associated inflammation and comorbidities. Increased plasma levels of interferon-α (IFN-α), interleukin-1 (IL-1), IL-6, IL-18, and tumor necrosis factor-α (TNF-α) (20, 21) cause activation and apoptosis of immune cells (20–22) or alter the homeostatic levels and functionality of the IL-17– and IL-22–producing lymphocytes (23), leading to mucosal inflammation and loss of integrity of the epithelial barrier (23, 24). Consequently, microbial products translocate into the general circulation, directly stimulating the immune system and boosting inflammation (12, 13, 25, 26). The importance of this mechanism of HIV/SIV disease progression is supported by the observation that microbial translocation and systemic inflammation are prevented in African nonhuman primate (NHP) SIV hosts, which maintain mucosal barrier integrity throughout infection (14, 27) through regenerative wound healing mechanisms (28, 29).

Meanwhile, virus replication is central to the direct stimulation of T cell activation (30). Immune suppression induced during the terminal HIV infection can trigger reactivation of opportunistic viruses otherwise controlled by a healthy immune system (31), contributing to immune activation through virus-infected cell recognition by antigen-specific T and B cells. The viral products of both HIV and opportunistic viruses can also boost inflammation by binding to pattern recognition receptors (30). T cell immune activation and apoptosis can be further boosted by inflammatory cytokines (3).

Inflammation and T cell immune activation are closely interconnected, as demonstrated by the observation that increased plasma levels of bacterial products associate with increased turnover of central memory CD4^+^ T cells (6), which contribute to the depletion of other mucosal CD4^+^ T cell subsets (13, 25) during pathogenic HIV/SIV infections (4).

We have previously provided multiple lines of evidence supporting a key role for alterations of the gut integrity and the subsequent microbial translocation in driving disease progression to AIDS. Through direct lipopolysaccharide (LPS) administration, we induced systemic inflammation and hypercoagulation in chronically SIV-infected African green monkeys (AGMs) (9, 32). Conversely, microbial translocation blockade with sevelamer transiently reduces acute and postacute systemic inflammation and hypercoagulation in pathogenic SIV infection (15). Direct mucosal damage with dextran sulfate (DSS) administration to uninfected macaques or chronically SIV-infected AGMs induced systemic inflammation, T cell immune activation, and hypercoagulation, similar to those observed in chronically SIV-infected macaques (33).

However, these studies could not assess the relative contribution of gut integrity or T cell immune activation as key mechanisms preventing disease progression in natural hosts of SIV, and conversely, the main driver of HIV/SIV disease progression. Therefore, here we dissected the in vivo mechanisms through which AGMs are able to resolve T cell immune activation and inflammation when transitioning from acute-to-chronic SIV infection. We report that, in spite of the multifactorial nature of the persistent T cell activation and inflammation in chronic HIV/SIV infection, mucosal barrier integrity appears to be the key driver of the control of chronic inflammation, comorbidities, and disease progression. Our results suggest that simply tackling T cell immune activation and systemic inflammation will likely not be effective for controlling HIV disease progression and that interventions should instead target the causes of the systemic inflammation and immune activation and hence attempt to restore gut integrity, the likely main driver of HIV disease progression.

## Results

### Study design and clinical data.

We dissociated T cell immune activation from intestinal mucosal integrity by inducing a partial depletion of the Tregs, using Ontak (Denileukin Diftitox, Ligand Pharmaceutical), an engineered immunotoxin (currently discontinued) that combines IL-2 with diphtheria toxin (DT). Ontak binds to the IL-2 receptor CD25, a molecule highly expressed by Tregs. As such, a significant fraction of Tregs expressing CD25 is targeted by the drug, and DT selectively kills them. Note that for in vivo Treg depletion, we cannot target the best Treg marker, FoxP3, which is intracellular.

We administered Ontak to 4 adult AGMs intravenously, at a dose of 15 μg/kg, for 5 consecutive days, starting 2 days prior to SIVsab92018 infection. This administration regimen was based on pharmacokinetics studies in macaques (34). Three rounds of treatment were performed (initiated at –2, 32, and 53 days postinfection, dpi), and Ontak was well tolerated by AGMs, with no grade II, III, and IV adverse events and no clinical sign of autoimmunity being observed during the follow-up. We also included 5 historical control AGMs, in which SIV infection followed its natural course and that were followed in the same conditions as the Ontak-treated AGMs. None of the Ontak-treated or control AGMs showed signs of SIV disease progression during a 166-day follow-up. Blood, superficial lymph nodes (LNs), and intestinal biopsies were collected from all AGMs at multiple matched time points during the follow-up.

### Ontak administration induces significant Treg depletion in AGMs that persists beyond treatment, throughout the follow-up.

**Figure 1:**
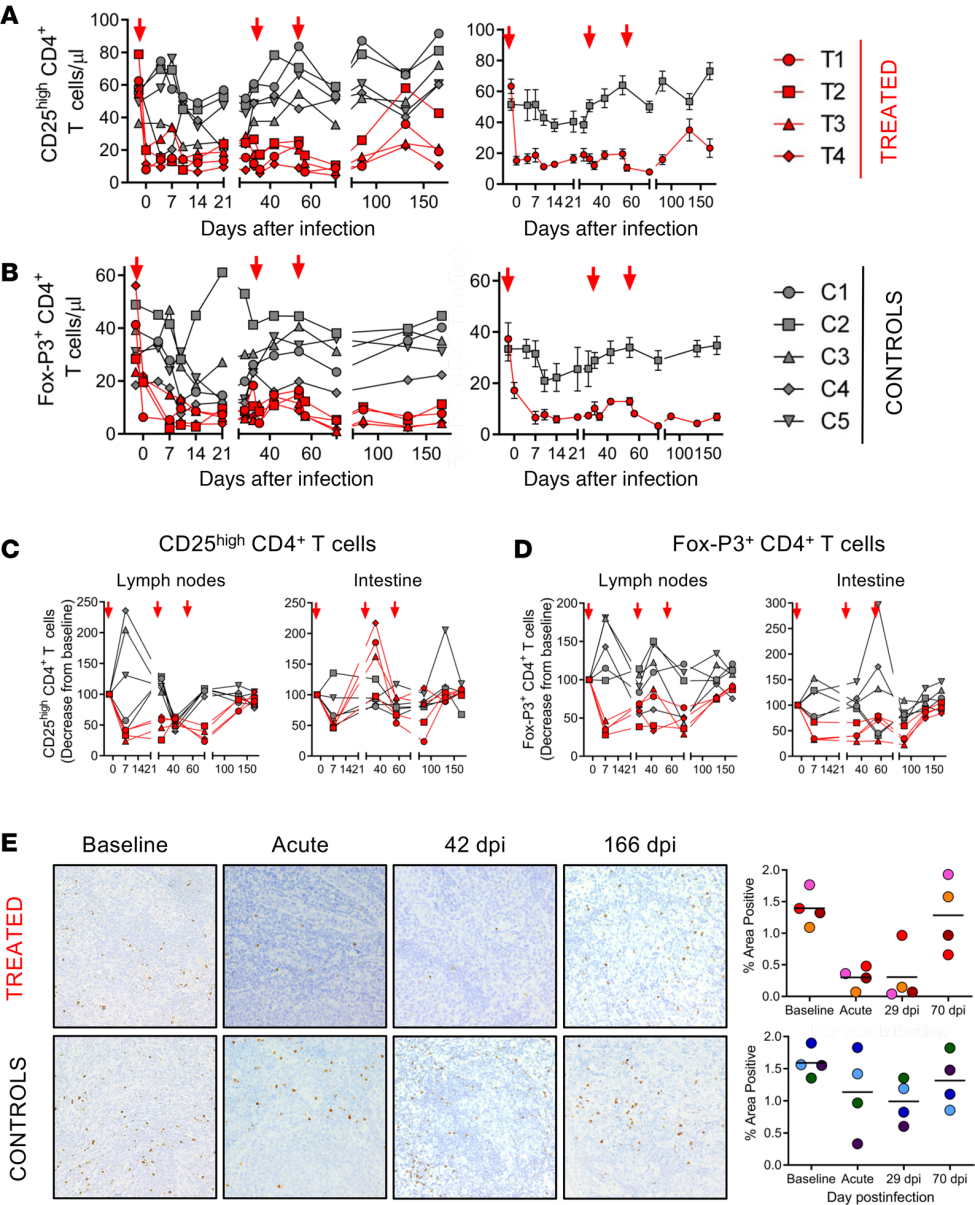
Ontak administration to SIV-infected AGMs depletes Tregs. (**A**) Comparison between CD25^hi^CD4^+^ T cell dynamics in acutely SIV-infected AGMs receiving Ontak (*n* = 4, red) and untreated controls (*n* = 5, gray). (**B**) Comparison between FoxP3^+^CD4^+^ T cell dynamics in acutely SIV-infected AGMs receiving Ontak (*n* = 4, red) and untreated controls (*n* = 5, gray). Shown are both the individual levels in treated versus untreated AGMs (left panels) and the average values for each group and standard error of means (right panels). Time points of Ontak treatment initiation (–2, 32, and 53 dpi) are marked by red arrows. Treg depletion in the lymph nodes (LNs) (left panels) and intestinal biopsies (right panels) after Ontak administration, as illustrated by the dynamics of the (**C**) CD25^hi^CD4^+^ T cells and (**D**) FoxP3^+^CD4^+^ T cells. (**E**) Flow cytometry data were validated by immunohistochemistry, showing a trend for FoxP3^+^ cell depletion in the LNs of the Ontak-treated AGMs compared with controls. Representative images (original magnification, 50×) of the LNs collected prior to infection, during the acute infection, at the setpoint (42 dpi), and during the chronic infection (166 dpi) and stained for FoxP3 (brown) in treated AGMs (*n* = 4) versus controls (*n* = 4). Quantification of the percentage area of the LNs positive for FoxP3.

Circulating CD25^hi^CD4^+^ T cells were depleted both in terms of absolute counts (Figure 1A) and as a fraction of CD4^+^ T cells (Supplemental Figure 1A; supplemental material available online with this article; https://doi.org/10.1172/jci.insight.161111DS1). Depletion was rapid and massive, involving 75%–85% of the circulating CD25^hi^CD4^+^ T cells 3 days after treatment initiation, from an average of 63 ± 5 CD25^hi^CD4^+^ T cells/μL preinfection to an average of 15 ± 3 CD25^hi^CD4^+^ T cells/μL at 2 dpi. Conversely, no relevant changes in the CD25^hi^CD4^+^ T cell counts occurred in controls: 51 ± 4 CD25^hi^CD4^+^ T cells/μL preinfection and 52 ± 11 CD25^hi^CD4^+^ T cells/μL at 4 dpi. The magnitude of circulating CD25^hi^CD4^+^ T cell depletion further increased with each treatment round (Figure 1A), and the CD25^hi^CD4^+^ T cells remained depleted throughout the follow-up (Figure 1A). As such, at the end of follow-up, 166 dpi and >100 days after the last round of Ontak treatment, CD25^hi^CD4^+^ T cells were still significantly lower in Ontak-treated AGMs compared with controls (*P* = 0.016) (Figure 1A).

A more limited CD25^hi^CD4^+^ T cell depletion (40%–50%) occurred in LNs (Figure 1C). In the gut, an initial expansion of the CD25^hi^CD4^+^ T cells, likely triggered by IL-2 DT-induced immune activation, was followed by a relatively modest depletion (25%–30%) of this cellular subset (Figure 1C). Conversely, CD25^hi^CD4^+^ T cells transiently increased in the LNs and intestine of controls. Therefore, we concluded that Ontak markedly depleted CD25^hi^CD4^+^ T cells.

An issue of Treg depletion with Ontak is that CD25 is not a strictly Treg-specific marker, yet the Treg-specific marker FoxP3 cannot be directly therapeutically targeted. Therefore, to directly evaluate Ontak’s impact on Tregs, we monitored the FoxP3^+^CD4^+^ T cells both by flow cytometry in periphery (Figure 1B), LNs, and gut (Figure 1D) and by IHC in the LNs (Figure 1E). As shown in Figure 1B, circulating FoxP3^+^CD4^+^ Tregs were substantially depleted in the acutely SIV-infected AGMs receiving Ontak (Figure 1B), from 37 ± 7 FoxP3^+^CD4^+^ T cells/μL preinfection to 7 ± 3 FoxP3^+^CD4^+^ T cells/μL at 6 dpi, i.e., a >80% depletion. As for the CD25^hi^CD4^+^ T cells, the FoxP3^+^CD4^+^ Tregs showed a trend toward transient rebound at 21 days after treatment initiation, which was reversed by additional administrations of the immunotoxin (Figure 1B).

Conversely, while a limited depletion of FoxP3^+^CD4^+^ T cells transiently occurred in controls during the acute SIV infection, the circulating FoxP3^+^CD4^+^ Tregs rebounded to virtually preinfection levels during the follow-up (Figure 1B), similar to CD25^hi^CD4^+^ T cells (Figure 1A).

A more limited FoxP3^+^CD4^+^ T cell depletion (close to 50%) occurred in the LNs (Figure 1D, left) and in the gut (Figure 1D, right), where over 50% of FoxP3^+^CD4^+^ T cells were depleted throughout the follow-up. Treg depletion in tissues persisted for up to 100 days after the last Ontak treatment. Again, this was different from the controls, in which FoxP3^+^CD4^+^ T cells transiently increased in the LNs and intestine. As such, Ontak markedly depleted FoxP3^+^CD4^+^ T cells.

IHC also showed a trend of reduction of FoxP3^+^ cells in the LNs during acute and postacute SIV infection in the AGMs receiving Ontak (Figure 1E) and virtually no changes in controls.

### Ontak minimally influences total CD8^+^ T cells.

**Figure 2:**
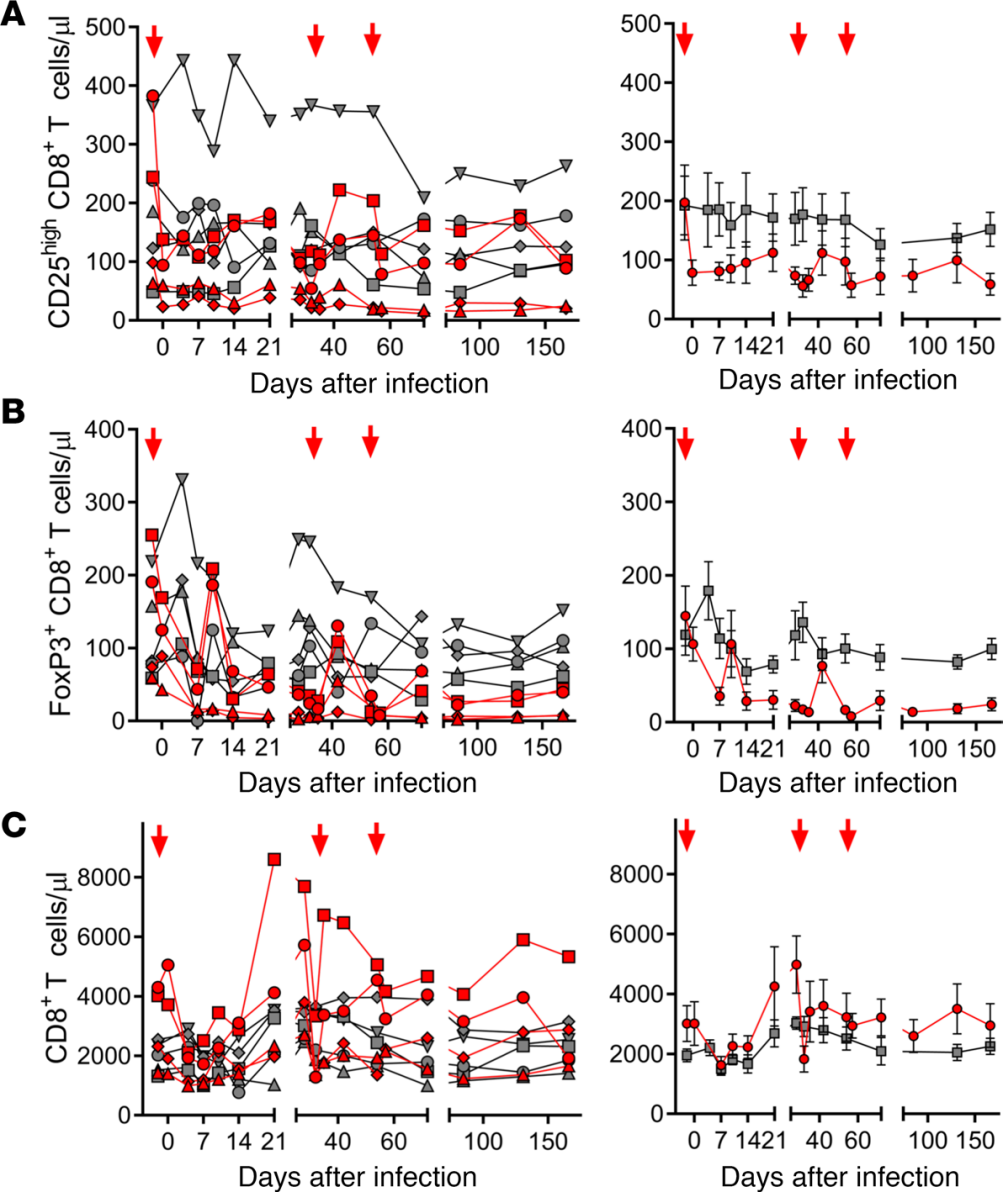
Ontak administration to SIV-infected AGMs has a minimal impact on the CD8^+^ T cell counts. (**A**) Comparison between CD25^hi^CD8^+^ T cell dynamics in SIV-infected AGMs receiving Ontak (*n* = 4, red) and untreated controls (*n* = 5, gray). (**B**) Comparison between FoxP3^+^CD8^+^ T cell dynamics in SIV-infected AGMs receiving Ontak (*n* = 4, red) and untreated controls (*n* = 5, gray). (**C**) Comparison between changes in the total CD8^+^ T cell population after Ontak administration in SIV-infected AGMs receiving Ontak (*n* = 4, red) and untreated controls (*n* = 5, gray). Shown are both the individual levels in treated versus untreated AGMs (left panels) and the average values for each group and standard error of means (right panels). Time points of Ontak treatment initiation (–2, 32, and 53 dpi) are shown by red arrows.

As Ontak targets CD25, which is expressed on the surface of multiple immune cell subsets, including CD8^+^ T cells, an impairment of cellular immune responses through depletion of CD8^+^ T cells expressing CD25 might be an unintended side effect (35). Depletion of CD25-expressing CD8^+^ T cells was roughly in the same range (40%–70%) as that of CD4^+^ T cells expressing this marker (Figure 2A), i.e., from 197 ± 73 CD25^hi^CD8^+^ T cells/μL preinfection to 81 ± 17 CD25^hi^CD8^+^ T cells/μL by 2 dpi (Figure 2A). This resulted in a 75% depletion of the FoxP3^+^CD8^+^ T cells (Figure 2B), from 145 ± 47 FoxP3^+^ CD8^+^ T cells/μL preinfection, to 36 ± 14 FoxP3^+^ CD8^+^ T cells/μL at 2 dpi. However, the impact of Ontak on the overall CD8^+^ T cell population was only moderate (Figure 2C), because the frequency of CD8^+^ T cells expressing FoxP3 was less than 5% (Figure 2C). Interestingly, a boost of CD8^+^ T cells occurred after each administration of Ontak, which resulted in an overall increase of the CD8^+^ T cell counts in the Ontak-treated group compared with controls (Figure 2). Importantly, Ontak did not hinder the SIV-specific cellular immune responses (Supplemental Methods and Supplemental Figures 2 and 3). Overall, the dynamics of the CD8^+^ T cell counts were very similar in Ontak-treated AGMs and controls.

### Ontak induces systemic and mucosal T cell proliferation and persistent activation throughout the acute and early chronic SIV infection, beyond the transition from acute to chronic infection.

**Figure 3:**
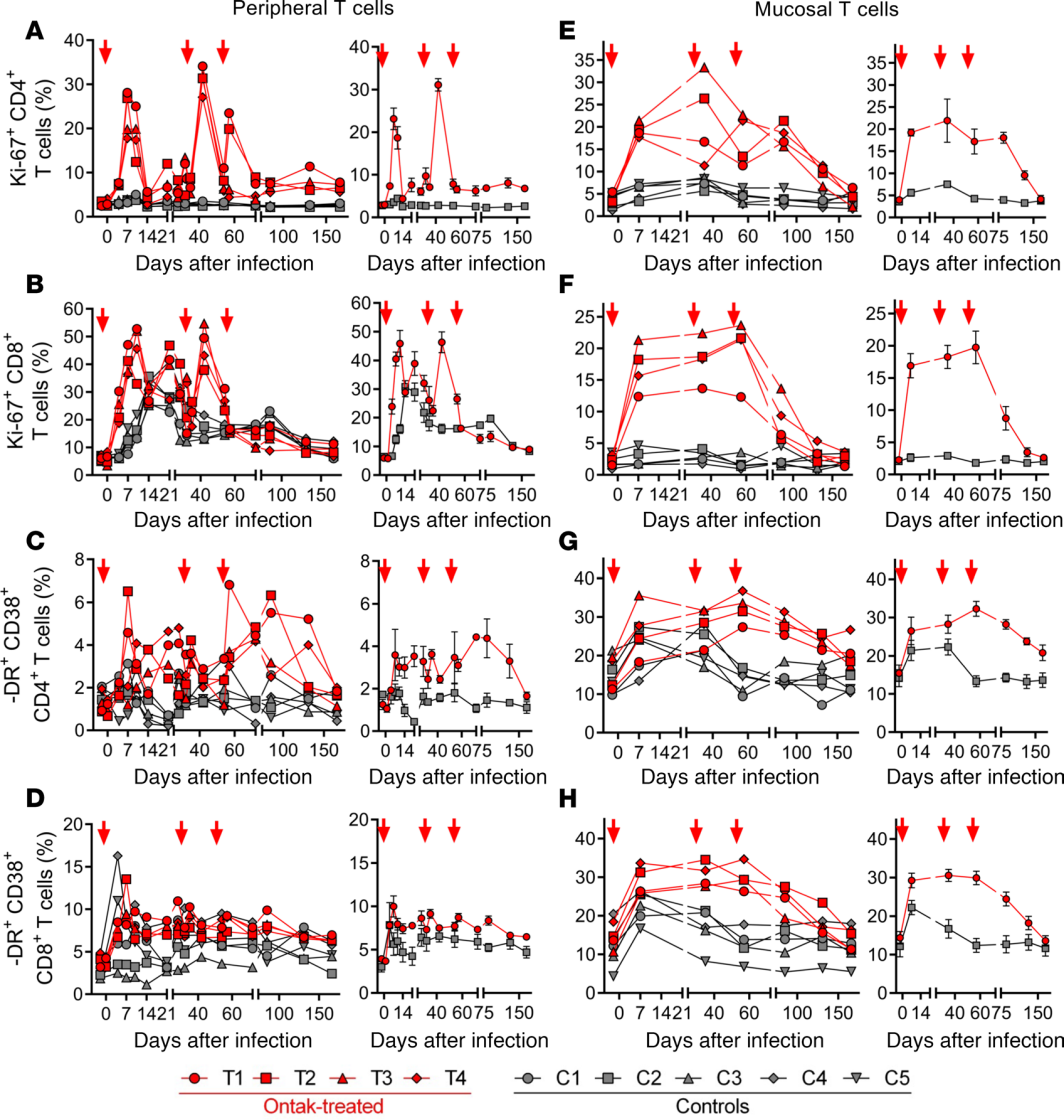
Ontak administration to SIV-infected AGMs ablates the resolution of T cell activation at the transition from acute to chronic infection. Substantial differences were observed between SIV-infected AGMs receiving Ontak (*n* = 4, red) and untreated controls (*n* = 5, gray) with regard to Ki-67 expression by peripheral CD4^+^ T cells (**A**) and CD8^+^ T cells (**B**) and CD38 and HLA-DR expression by peripheral CD4^+^ T cells (**C**) and CD8^+^ T cells (**D**). Massive increases in the expression of the T cell proliferation and activation marker were observed in the SIV-infected AGMs receiving Ontak (*n* = 4, red) compared with the untreated controls (*n* = 5, gray) with regard to Ki-67 expression by mucosal CD4^+^ T cells (**E**) and CD8^+^ T cells (**F**) and CD38 and HLA-DR expression by the mucosal CD4^+^ T cells (**G**) and CD8^+^ T cells (**H**). Shown are both the individual levels in treated versus untreated AGMs (left panels) and the average values for each group and standard error of means (right panels). Time points of Ontak treatment initiation (–2, 32, and 53 dpi) are shown by red arrows.

A massive increase in circulating CD4^+^ T cell proliferation occurred after each round of Ontak, as illustrated by the significant increases in the frequency of circulating T cells expressing the proliferation marker Ki-67 (*P* < 0.005 for CD4^+^ and CD8^+^, in the acute phase and early chronic infection during Ontak effects) in Ontak-treated versus control AGMs (Figure 3, A and B). Since these increases may result from homeostatic T cell proliferation in an activation-independent manner occurring in response to the loss of regulatory and total T cells, we monitored additional markers of immune activation, such as HLA-DR and CD38. Ontak induced persistent increases of circulating T cells expressing CD38 and HLA-DR. These increases involved CD4^+^ T cells (*P* = 0.0009, acute, and *P* = 0.0004, chronic), and to a lesser extent CD8^+^ T cells (*P* = 0.057 and *P* = 0.09) (Figure 3, C and D), and persisted throughout the follow-up, beyond the acute-to-chronic stage of infection (Figure 3, C and D), when immune activation is usually controlled in natural hosts of SIVs (36–38), as seen in controls (Figure 3).

The increased activation and proliferation of peripheral CD4^+^ T cells were significant but limited. Therefore, we validated these results by monitoring the expression of the same parameters in T cells isolated from intestinal biopsies. Dramatic and persistent increases of Ki-67 expression of mucosal T cells occurred in Ontak-treated AGMs, when compared with controls. Ki-67 expression increased (as early as 7 dpi), and this difference was maintained throughout the follow-up on both CD4^+^ T cells (*P* < 0.0001) (Figure 3E) and CD8^+^ T cells (*P* = 0.0001) (Figure 3F), being normalized only at necropsy.

Massive and persistent increases of mucosal T cell activation were also found when measuring CD38 and HLA-DR expression on CD4^+^ (*P* = 0.0008) and CD8^+^ (*P* < 0.0001) T cells (Figure 3, G and H). The differences between Ontak-treated AGMs and controls were attenuated only at the end of the follow-up, well beyond the acute-to-chronic stage of infection (Figure 3).

These results indicate that Ontak persistently increased systemic and mucosal T cell activation in acutely SIV-infected AGMs, ablating the resolution of immune activation that usually occurs at the transition from acute to chronic infection.

### Ontak did not change the levels of macrophages in the LNs or increase their activation status.

Chronic macrophage activation by microbial products translocated following gut mucosal damage is central to the pathogenesis of HIV/SIV disease (9, 12, 39, 40). Therefore, we assessed the impact of Treg depletion on macrophages (CD14^+^) in LNs (Supplemental Figure 4) (41). The fraction of CD14^+^ macrophages significantly increased in Ontak-treated AGMs throughout the follow-up (*P* = 0.048) (Supplemental Figure 4). However, the activated macrophages (CD80^+^CD86^+^) did not increase in Ontak-treated AGMs, being even decreased at several time points during the follow-up (*P* = 0.0002) (Supplemental Figure 4), probably as a result of the Treg depletion decreasing the levels of IL-10 and TGF-β, 2 cytokines involved in macrophage activation (42). We concluded that our intervention dissociated chronic T cell activation from macrophage activation, and we further assessed its impact on the levels of systemic inflammation.

### Ontak administration to SIV-infected AGMs induces only transient increases of the levels of systemic inflammation, which do not persist between treatments.

**Figure 4:**
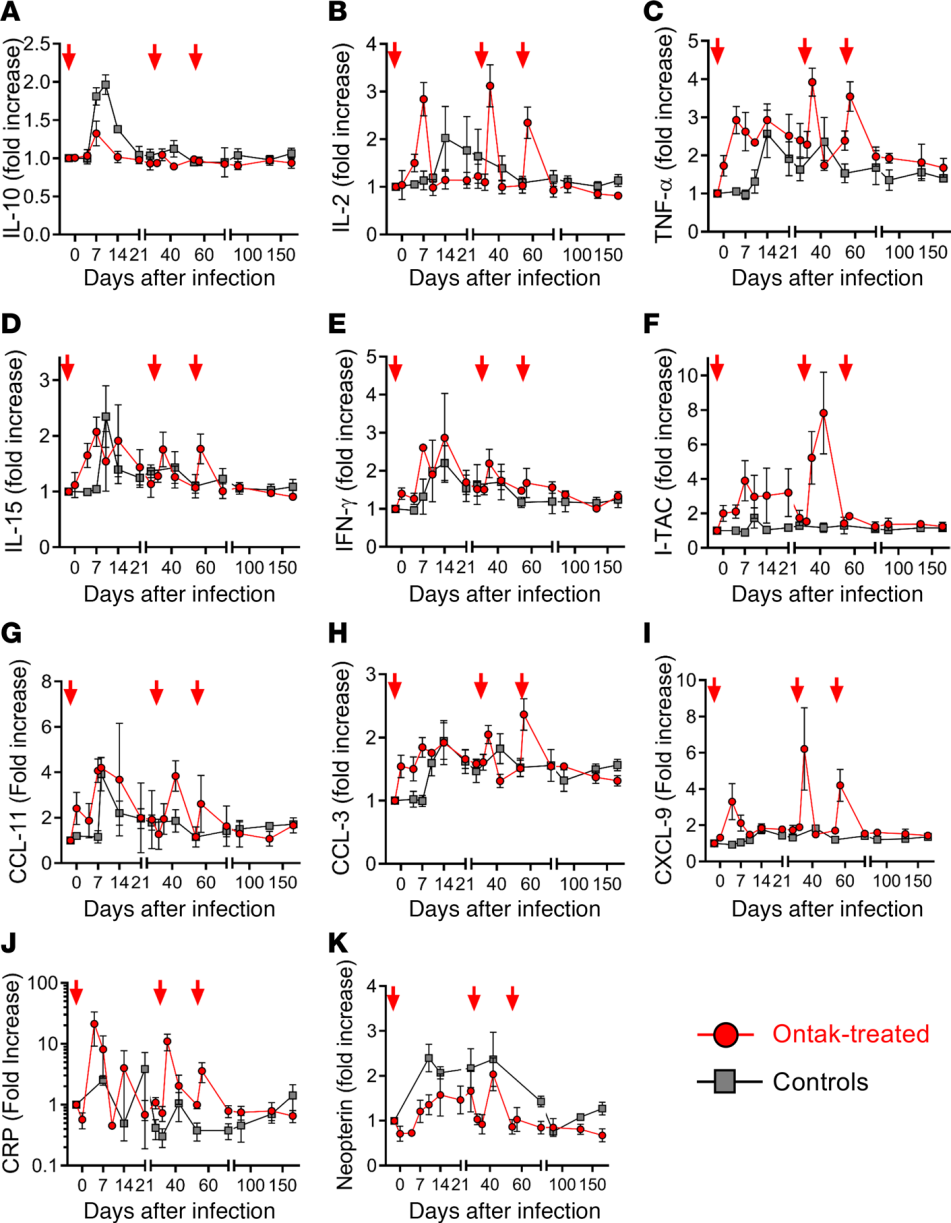
Ontak administration to SIV-infected AGMs induced only transient spikes in the inflammatory biomarkers. Shown are the very transient increases, or no increases, observed in Ontak-treated AGMs (*n* = 4, red) compared to untreated controls (*n* = 4, gray) for IL-10 (**A**), IL-2 (**B**), TNF-α (**C**), IL-15 (**D**), IFN-γ (**E**), interferon-inducible T cell alpha chemoattractant (I-TAC) (**F**), CCL-11 (eotaxin) (**G**), CCL-3 (MIP-1α) (**H**), and CXCL-9 (MIG) (**I**), as well as for plasma biomarkers of inflammation predictive for HIV disease progression and death: C-reactive protein (CRP) (**J**) and neopterin (**K**). Shown are the average values for each group and standard error of means. Calculations have been done as fold increases from the average of 3 preinfection baseline values. Time points of Ontak treatment initiation (–2, 32, and 53 dpi) are illustrated by red arrows.

By comparing the plasma levels of a variety of cytokines/chemokines IL-10 (Figure 4A), IL-2 (Figure 4B), TNF-α (Figure 4C), IL-15 (Figure 4D), IFN-γ (Figure 4E), and I-TAC (Figure 4F); chemokines CCL-11 (Figure 4G), CCL-3 (Figure 4H), and CXCL9 (Figure 4I); and biomarkers of inflammation with predictive value for HIV/SIV disease progression and death, such as CRP (Figure 4J) and neopterin (Figure 4K), between treated and untreated AGMs, we showed that Ontak did not induce major persistent increases in systemic inflammation. The levels of the antiinflammatory IL-10 were lower in the Treg-depleted AGMs than in controls (Figure 4A), especially during acute SIV infection, but likely above the threshold that would lead to macrophage activation. These results were supported by the dynamics of neopterin, a soluble marker produced by macrophages, that also showed a trend toward lower levels in the Ontak-treated AGMs (Figure 4K). In contrast to T cell activation markers, which were increased throughout follow-up (Figure 3), the increases of the inflammatory markers were minimal and transient, mostly spiking after each Ontak administration before going back to the baseline levels, which were maintained in controls throughout the chronic infection (Figure 4).

As such, our results revealed a dissociation between T cell activation (consistently increased in AGMs receiving Ontak), and inflammation, which did not occur in the Ontak-treated AGMs (where only transient spikes of the inflammatory markers occurred with every round of Ontak administration, followed by a rapid return to nearly normal values between treatments) (Supplemental Figure 4 and Figure 4). Thus, we concluded that the mucosal barrier was probably maintained in Ontak-treated AGMs.

### Ontak boosts SIV replication and delays postacute CD4^+^ T cell restoration in AGMs.

**Figure 5:**
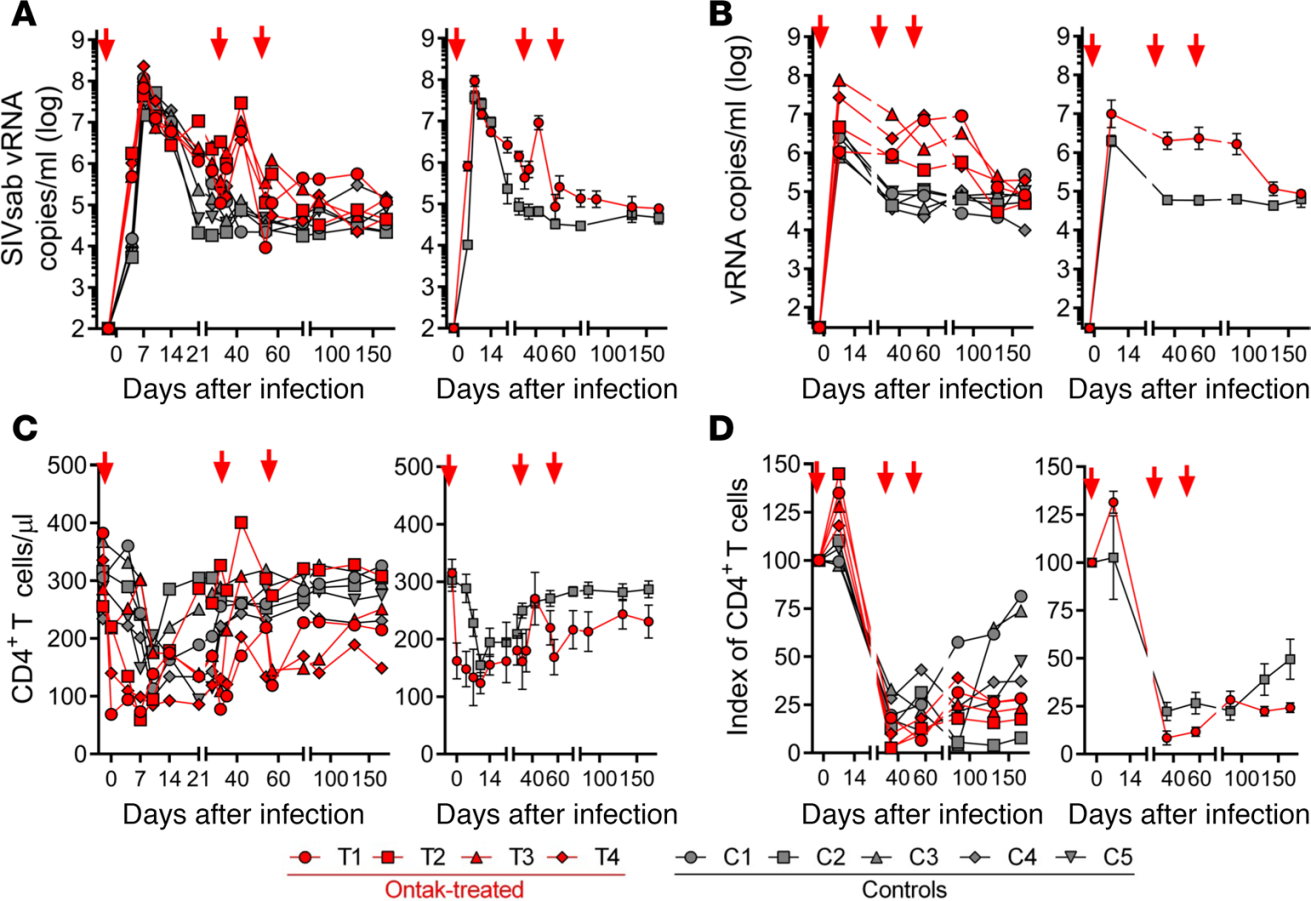
Ontak administration to SIV-infected AGMs induces minimal changes in key biological parameters of SIV pathogenesis. (**A**) Slight increases in the viral loads were observed in the SIV-infected AGMs receiving Ontak (*n* = 4, red) compared with untreated controls (*n* = 5, gray). (**B**) More prominent differences in the levels of SIV RNA between Ontak-treated AGMs and controls were observed in the gut. (**C**) Depletion of peripheral CD4^+^ T cells was more robust (albeit not significantly different) in the SIV-infected AGMs receiving Ontak (*n* = 4, red) compared with untreated controls (*n* = 5, gray). In the intestine, mucosal CD4^+^ T cell depletion was more pronounced and more prolonged in the SIV-infected AGMs receiving Ontak (*n* = 4, red) compared with untreated controls (*n* = 5, gray) (**D**). Shown are both the individual levels in treated versus untreated AGMs (left panels) and the average values for each group and standard error of means (right panels). Time points of Ontak treatment initiation (–2, 32, and 53 dpi) are shown by red arrows. vRNA, viral RNA.

We next assessed the consequences of persistently increased immune activation on the pathogenesis of SIV infection. Ontak boosted viral replication, with VLs becoming detectable earlier and at higher levels in Ontak-treated AGMs than in controls (Figure 5A). Although pVLs peaked at similar levels (around 10^8^ viral RNA [vRNA] copies/mL of plasma) in both treated and untreated animals (*P* = 0.09 for acute VL) (Figure 5A), the postpeak and early chronic plasma VLs were approximately 1 log higher in Ontak-treated AGMs than in controls (*P* = 0.0004). These differences were resolved by the end of follow-up (166 dpi), when VLs were no longer significantly different between the 2 groups (*P* = 0.41).

The differences between the Ontak-treated AGMs and controls were more prominent in the gut, where Ontak administration resulted in higher cell-associated vRNA (CA-vRNA) loads throughout the first 100 dpi (Figure 5B). Higher peak VLs were observed in the gut in the Ontak-treated AGMs compared with controls (7 ± 0.4 log versus 6.3 ± 0.16 log CA-vRNA, respectively) (Figure 5B). Setpoint and early chronic VLs were also higher by about 1–1.5 log CA-vRNA in the gut of the Ontak-treated AGMs, compared with controls (*P* < 0.0001) (Figure 5B). As observed for pVLs, at necropsy, after the effect of Ontak wore off, CA-vRNA in the gut was similar in both groups (*P* > 0.9).

Upon SIV infection, the levels of circulating CD4^+^ T cells decreased during acute infection (8–10 dpi) in both Ontak-treated (from 315 ± 28 CD4^+^ T cells/μL to 124 ± 21 CD4^+^ T cells/μL) and control AGMs (from 303 ± 22 CD4^+^ T cells/μL to 155 ± 33 CD4^+^ T cells/μL) (Figure 5C). During chronic infection, a delay of CD4^+^ T cell restoration was observed in Ontak-treated AGMs, resulting in slightly lower levels of circulating CD4^+^ T cell counts than in controls; yet these differences did not reach statistical significance (*P* = 0.19). Each subsequent administration of Ontak transiently boosted the levels of peripheral CD4^+^ T cell counts (Figure 5C).

Mucosal CD4^+^ T cells were massively depleted in both Ontak-treated AGMs and controls (Figure 5D). However, in spite of a slightly more prominent acute depletion and a more limited and delayed restoration of the mucosal CD4^+^ T cells, there were no relevant differences between the 2 groups (Figure 5D). Of note, at the mucosal sites, the initial administration of Ontak resulted in a significant early (7 dpi) increase of mucosal CD4^+^ T cells when compared with controls (*P* = 0.016), probably as a result of the massive CD4^+^ T cell activation and proliferation induced by Ontak, leading to overexpression of mucosal homing markers, such as CCR5 (43, 44).

Despite resulting in increased viral replication and slightly more severe and prolonged acute CD4^+^ T cell depletion, ablation of the resolution of T cell activation at the transition from acute to chronic infection in the absence of mucosal damage did not significantly alter the major parameters of SIV infection, most notably disease progression, in a natural host of SIV.

### Dissociation of mucosal barrier integrity from persistent T cell activation in Ontak-treated AGMs.

We next assessed the impact of the partial Treg depletion with Ontak on the gut integrity to determine whether the mucosal barrier was maintained in the Ontak-treated AGMs, in spite of the persistent T cell activation induced by Treg depletion, and therefore that microbial translocation and chronic inflammation that fuel T cell immune activation and disease progression (5, 13, 25) were averted in these animals.

**Figure 6:**
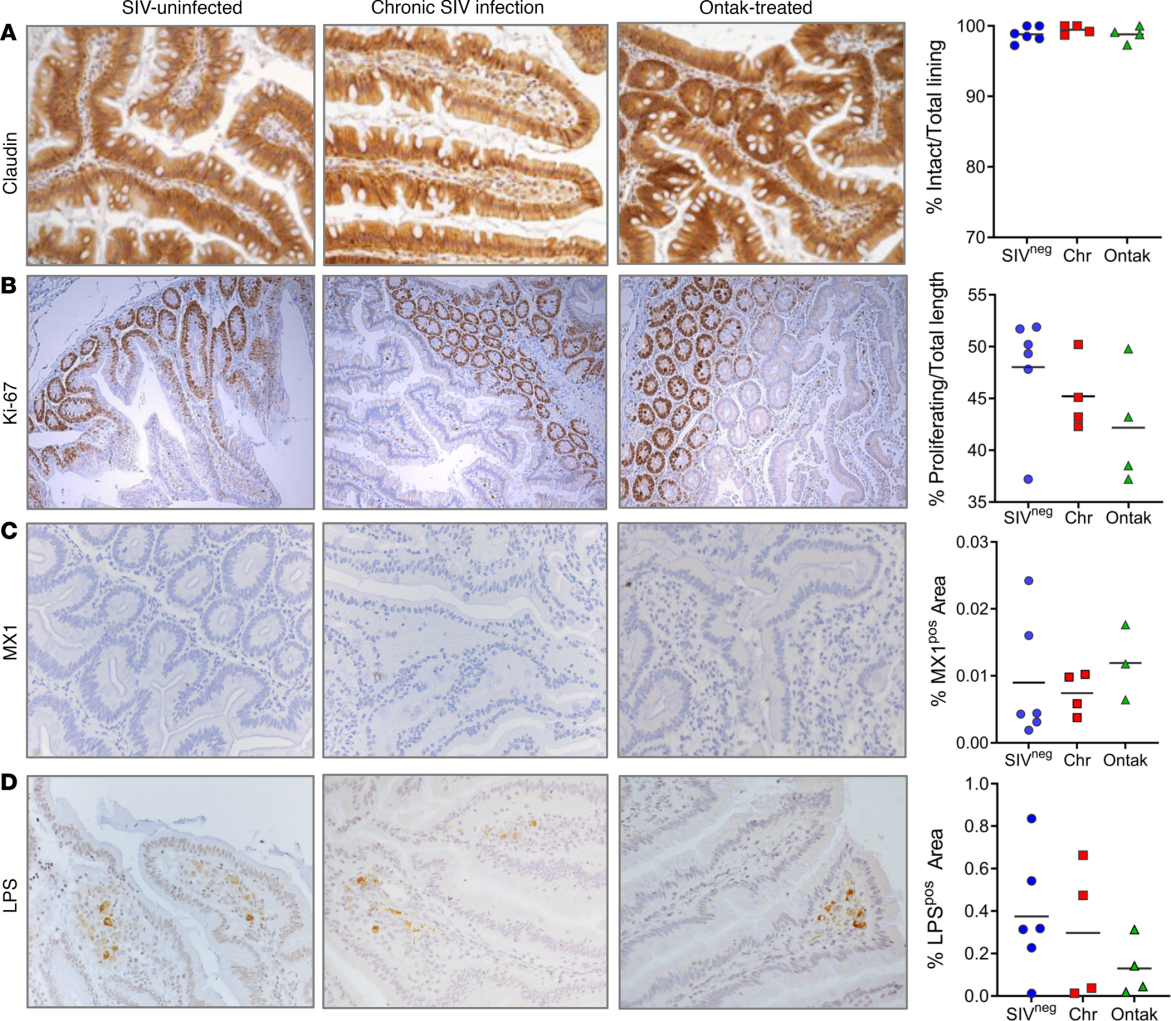
Ontak administration to SIV-infected AGMs does not result in alterations of the integrity of the intestinal mucosal barrier. (**A**) Representative images (original magnification, 200×) of intestine stained for the tight junction protein claudin-3 (brown) as a marker for intact epithelial barrier in historically uninfected controls (*n* = 6), chronically SIV-infected untreated AGMs (*n* = 4), and Ontak-treated AGMs (*n* = 4) documenting no detectable damage and discontinuities of the epithelial barrier. Quantitative image analyses of the integral versus total epithelial lining in the intestine in uninfected controls, chronically SIV-infected untreated AGMs, and Ontak-treated AGMs failed to find any statistical difference (Mann-Whitney *U* test). (**B**) No significant difference in the ratio of proliferating cells (Ki-67^+^) in the crypts versus the total lengths of the crypts between uninfected controls, chronically SIV-infected untreated AGMs, and Ontak-treated AGMs. Representative images (original magnification, 100×) of intestine stained for the proliferative nuclear protein Ki-67 (brown). No significant change in frequency and location of Ki-67^+^ enterocytes. Quantitative image analysis of Ki-67 expression by enterocytes in historically uninfected controls (*n* = 6), chronically SIV-infected untreated AGMs (*n* = 4), and Ontak-treated AGMs (*n* = 4) finds no statistically significant increase of the ratio of proliferating cells (Ki-67^+^) in crypts versus the total lengths of the crypts (Mann-Whitney *U* test). (**C**) No significant change in frequency and location of Mx1^+^ enterocytes (original magnification, 200×) in historically uninfected controls (*n* = 6), chronically SIV-infected untreated AGMs (*n* = 4), and Ontak-treated AGMs (*n* = 3). (**D**) Representative images (original magnification, 200×) of intestine sections from uninfected controls, chronically SIV-infected untreated AGMs, and Ontak-treated AGMs stained for LPS core antigen (brown). Quantitative image analysis of the LPS levels within the lamina propria of the intestine in historically uninfected controls (*n* = 6), chronically SIV-infected untreated AGMs (*n* = 4), and Ontak-treated AGMs (*n* = 4) finds no statistically significant increase (Mann-Whitney *U* test). Uninfected controls: blue circles; chronically SIV-infected untreated AGMs: red squares; and Ontak-treated AGM: green triangles.

By using a combination of IHC, microscopy, and computer-aided quantification, we evaluated the gut epithelium for any signs of major perturbations following treatment with Ontak. We first directly assessed its overall integrity by staining histologically prepared sections for the tight junction protein claudin-3. Loss of claudin-3 in response to SIV infection indicates loss of mucosal integrity (1, 5), which triggers microbial translocation. Quantification of the ratio of intact versus damaged epithelium in the gut showed no significant damage or loss of epithelial continuity in either the chronically SIV-infected Ontak-treated AGMs or controls (Supplemental Figure 5A and Figure 6A). This is in stark contrast to pathogenic SIV infection of RMs, in which a massive loss of claudin-3 and major damage to the gut epithelium occurs during chronic infection (14). Quantitative image analysis from high-power entire intestinal tissue sections verified the lack of epithelial damage and maintenance of barrier integrity in both Ontak-treated AGMs and controls, similar to uninfected AGMs (Figure 6A and Supplemental Figure 5A).

After documenting that chronic SIV-infected Ontak-treated AGMs and controls present with virtually no damage to the gut epithelium, we assessed the effects of partial Treg depletion on epithelial cell proliferation. In RMs, pathogenic SIV infection results in a sharp increase in Ki-67 expression by epithelial cells in the crypts, as measured by the length of the Ki-67^+^ cells versus total crypt length (1). Yet, no such increase was observed during chronic SIV infection in either Ontak-treated AGMs or controls (Supplemental Figure 5B). In fact, epithelial Ki-67 expression decreased in both groups. While this decrease was minor (~10%–15%), it was statistically significant for both Ontak-treated AGMs (*P* = 0.0162) and controls (*P* = 0.0281) (Figure 6B). No significant difference could be established between Ontak-treated AGMs and controls, indicating that Ontak does not significantly alter the proliferation status of gut epithelial cells compared with SIV infection alone.

We next verified that we successfully dissociated mucosal damage/integrity from persistent systemic T cell activation in SIV-infected AGMs, by using IHC and quantitative image analysis tools to measure the dynamics of the interferon-inducible protein Mx1, a marker of gut inflammation (45, 46). Mx1 dynamics showed no increase in the inflammatory responses in Ontak-treated AGMs compared to controls (Figure 6C) and no significant increase from the baseline levels in either group, verifying that our intervention did not induce significant mucosal inflammation and gut damage.

Microbial translocation is the key consequence of the mucosal injury induced by pathogenic HIV/SIV infections (13, 14, 47). The natural hosts of SIV have the ability to keep microbial translocation at bay (38, 48). Therefore, we monitored the levels of microbial translocation by staining with a monoclonal antibody (mAb) against LPS core antigen. The levels of microbial products did not increase in the lamina propria of either SIV-infected Ontak-treated AGMs or controls as compared to uninfected animals (Figure 6D). Quantitative image analyses validated these results (Figure 6D).

**Figure 7:**
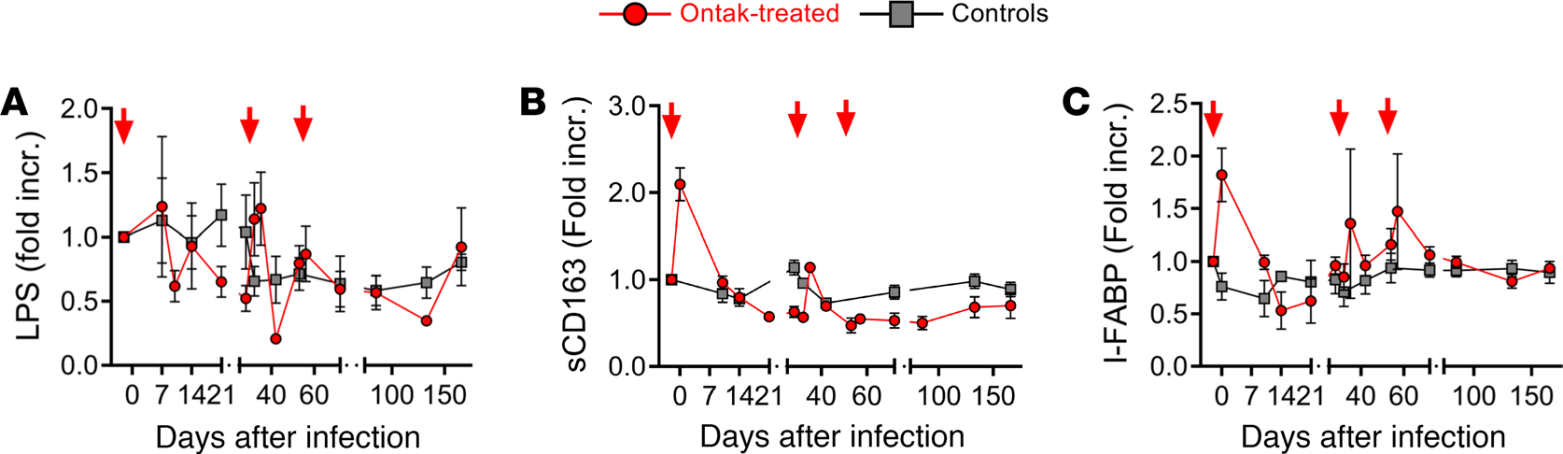
Ontak administration to SIV-infected AGMs does not result in significant and persistent changes of the soluble biomarkers of systemic microbial translocation and gut integrity. (**A**) No significant increase in the plasma LPS levels between Ontak-treated AGMs (*n* = 4, red) and controls (*n* = 4, gray). (**B**) No significant changes in the levels of sCD163, a marker of macrophage activation, between Ontak-treated AGMs (*n* = 4, red) and controls (*n* = 5, gray). (**C**) Testing the levels of I-FABP, a surrogate marker of mucosal integrity, showed only very transient increases in the Ontak-treated AGMs (*n* = 4, red), that superposed those of the inflammatory markers. Shown are the average fold increases from the baseline for each group and standard error of means. Time points of Ontak treatment initiation (–2, 32, and 53 dpi) are shown by red arrows.

To relate microbial translocation in the gut of Ontak-treated AGMs or controls to systemic microbial translocation, we measured the plasma levels of LPS and showed neither a significant increase from the baseline nor a difference between the Ontak-treated AGMs and controls (*P* > 0.5 for all time periods) (Figure 7A). Furthermore, testing of sCD163 (Figure 7B), a soluble marker of macrophage activation, paralleled the results of the LPS testing, increasing only very transiently at the time of the first Ontak administration (Figure 7B), pointing again to preservation of gut integrity in AGMs and the fact that our intervention dissociated it from systemic T cell activation.

Finally, to verify the IHC results and document maintenance of the gut integrity in Ontak-treated AGMs, we used ELISA to monitor the plasma levels of intestinal fatty acid–binding protein (I-FABP), a protein released by necrotic enterocytes, which is used as a biomarker of intestinal damage (46). I-FABP testing showed only transient increases in Ontak-treated AGMs, corresponding to Ontak administration, and no relevant changes over baseline in controls (Figure 7C), and was associated with the spikes in other inflammatory biomarkers. This demonstrates again that inflammation is related to the gut damage and not to systemic T cell activation.

We therefore concluded that, since the Ontak treatment did not damage the gut, the preservation of mucosal integrity rather than control of immune activation is the main determinant of the lack of disease progression in natural hosts of SIV.

### Chronic T cell activation induced by Ontak does not result in persistent increases in coagulation markers in the absence of gut damage in SIV-infected AGMs.

**Figure 8:**
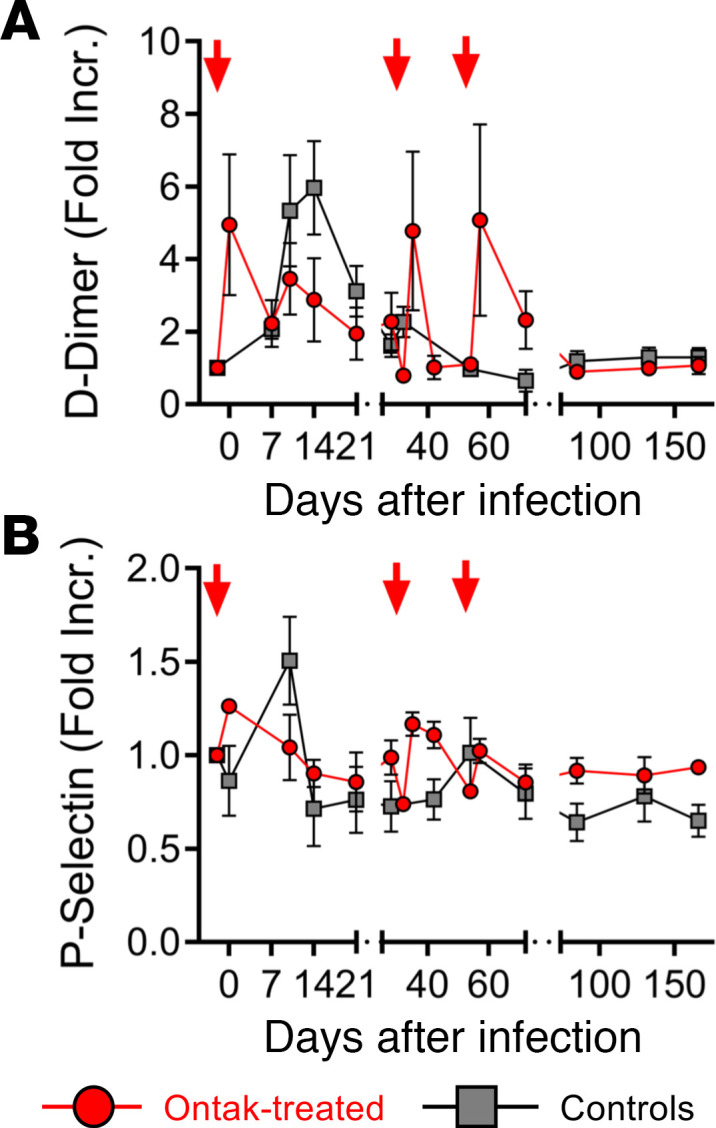
Ontak administration to SIV-infected AGMs induces minimal changes in key soluble biomarkers of hypercoagulation in the absence of systemic inflammation. (**A**) Transient increases in the levels of the D-dimer in Ontak-treated AGMs (*n* = 4, red), which were not significantly different from those in controls (*n* = 5, gray); the transient spikes corresponded to Ontak administration and the transient increases in biomarkers of inflammation. (**B**) No significant increases in the levels of the endothelial activation biomarker P-selectin between Ontak-treated AGMs (*n* = 4, red) and controls (*n* = 5, gray). Shown are the average fold increase from the baseline values for each group and standard error of means. Time points of Ontak treatment initiation (–2, 32, and 53 dpi) are shown by red arrows.

A hypercoagulable status has been associated with persistent gut dysfunction and increased inflammation characteristic of chronic HIV infection and is a very strong correlate of death in HIV-infected individuals and SIV-infected NHPs (7, 9). To further examine the impact of Ontak on the coagulation status, as a correlate of disease progression, we tested 2 coagulation biomarkers: D-dimer, the strongest predictor of lethality in HIV-infected individuals, and P-selectin, a marker of endothelial and platelet activation. D-dimer testing did not show significantly different dynamics between Ontak-treated AGMs and controls (*P* > 0.25). Note, however, that Ontak was associated with transient spikes of D-dimer levels (Figure 8A), simultaneous with the spikes in other inflammatory markers (Figure 4). Similarly, the levels of P-selectin also paralleled those of the inflammatory markers (Figure 8B). We concluded that an intermittent and transient inflammation in the absence of consistent mucosal intestinal damage in Ontak-treated AGMs was insufficient to trigger an increase in the coagulation markers associated with comorbidities and death in HIV and SIV pathogenic infections.

## Discussion

In this study we investigated the mechanisms of SIV disease progression (or lack thereof) in SIV-infected NHPs that are natural hosts of SIVs. We induced high and persistent levels of T cell immune activation in the absence of mucosal dysfunction and chronic inflammation in a model that naturally lacks SIV disease progression. In this way, we were able to dissect these 2 key pathogenic features of HIV infection and conclude that the integrity of the mucosal barrier and not the resolution of T cell activation at the transition from acute-to-chronic infection is the main correlate of the lack of disease progression in natural hosts of SIVs. By directly demonstrating for the first time to our knowledge that intestinal dysfunction is the main determinant of systemic inflammation and disease progression, our results have a substantial impact on understanding the outcome of HIV infection and point to the usefulness of therapeutic strategies aimed at preserving gut integrity to avoid progression to AIDS, comorbidities, and death.

HIV infection damages the mucosal barrier severely, triggering microbial translocation from the intestinal lumen into the gut mucosa and subsequently into the general circulation (25). Gut dysfunction occurs early during HIV infection and initiates a vicious cycle in which microbial translocation induces generalized inflammation and fuels T cell activation, virus replication, and cell death, which further enhance gut damage (5, 13).

While persistent GI tract barrier damage and microbial translocation are thought to continuously activate innate and adaptive immune responses in HIV/SIV infections (12), the causative relationship between intestinal dysfunction and HIV/SIV disease progression has never been proved. As such, more than a decade after the first release of the microbial translocation theory (13), there is still debate on whether microbial translocation is a cause or a consequence of the chronic T cell activation and inflammation in HIV-infected individuals (26, 49).

The main roadblock to our understanding of the role of intestinal dysfunction in the pathogenesis of AIDS is the lack of interventional tools to clearly demonstrate the role of the gut damage in disease progression. Severe intestinal dysfunction occurs early in HIV/SIV infection, concomitantly with or prior to every other key pathogenic features (i.e., the massive depletion and disruption of the homeostasis of CD4^+^ T cells), from which it cannot be dissociated. Interventions aimed at further increasing gut dysfunction and investigation of its impact on the key parameters of infection and disease progression cannot be undertaken without endangering the lives of HIV-infected individuals. Finally, damaging the gut in uninfected animals is not an option, as such an approach lacks virus replication, the key trigger of pathogenesis.

To dissect the contribution of mucosal dysfunction to HIV pathogenesis, we needed a model in which highly active lentiviral replication coexists with an intact gut. The natural hosts of SIV address all the requirements for such an intervention: high VLs, intact gut, no microbial translocation, and maintenance of baseline levels of inflammation and T cell activation throughout a nonprogressive SIV infection (8, 14, 29).

Through DSS administration to chronically SIV-infected AGMs, we previously damaged GI mucosa and induced colitis with elevated levels of plasma LPS, sCD14, and inflammation and increased viral replication (33). These changes persisted between the DSS rounds, showing that once gut damage occurs, its healing is difficult due to the feedback initiated by the gut pathology, which leads to gut permeability, microbial translocation, systemic T cell activation, and inflammation that fuel viral replication and gut dysfunction, maintaining this process, eventually leading to progression to AIDS (5).

Conversely, in a model of pathogenic SIV infection, we showed that LPS blockade in the gut transiently lowered microbial translocation and limited the levels of chronic inflammation and subsequent T cell activation, attenuating the key parameters of acute SIV infection (15), thus providing proof of the key role of microbial translocation in HIV/SIV disease progression.

Here, we performed an intervention aimed at ablating the control of T cell activation during chronic SIV infection in AGMs. Multiple studies have reported that the control of T cell activation at the passage from acute to chronic infection (36, 37, 50) is the main mechanism protecting natural hosts from disease progression (8, 15, 29, 51), together with a downregulation of the CD4 molecule by the helper cells that enter the memory pool (52, 53). Therefore, our goal was to maintain T cell activation beyond the passage from acute to chronic infection, thus abrogating a key factor reported to protect the natural hosts from disease progression (36, 37, 50). We have previously reported that Treg depletion with Ontak in chronically SIV-infected AGMs results in a massive activation of T cells that significantly boosts viral replication and mucosal T cell depletion, irrespective of the level of expression of the CD4 molecule (32). Here, Tregs were depleted prior to SIV infection and throughout the acute and postacute stages of infection. Our intervention prolonged T cell activation for over 100 days of infection, well beyond the transition from acute to chronic stage of SIV infection, maintaining an increased viral replication and delaying the postacute CD4^+^ T cell restoration. Yet, during more than 150 days of follow-up, Treg depletion did not significantly impact the natural history of SIV infection, and we did not observe any significant change in the biomarkers associated with disease progression or any significant change in the inflammatory and coagulation biomarkers predictive for comorbidities or death (7, 40). This was in stark contrast with our previous interventions, in which alteration of the mucosal barrier significantly impacted the natural history of SIV infection in its AGM host (33).

We therefore concluded that our intervention did not impact the outcome of SIV infection in AGMs because we did not alter the integrity of the mucosal barrier, which is normally maintained in natural hosts of SIVs throughout infection (27). Indeed, we directly assessed the impact of Ontak-induced prolonged T cell activation on the intestinal mucosa and failed to document any significant alteration of the mucosal integrity or enterocyte activation, any increase in the levels of local or systemic inflammation, or any microbial translocation. We concluded that by inducing an “irrelevant” T cell activation, we actually successfully dissociated persistent T cell activation from systemic inflammation induced by the mucosal damage characteristic of HIV/SIV infection. Through this intervention, we clearly demonstrated the key role the intestinal dysfunction plays in the pathogenesis of AIDS and HIV-associated comorbidities.

One may argue that the follow-up in this study was relatively short and that, had we maintained high levels of T cell activation for a longer period, we might have observed progression to AIDS. While this is a plausible scenario that cannot be dismissed, previous studies in which we directly induced mucosal lesions in SIV-infected AGMs provided clear indications of biological changes that had the potential to lead to both comorbidities and progression to AIDS within a much shorter time frame than the current study (33). Such biological changes were not seen here.

It can also be argued that the efficacy of Ontak is relatively poor, with only a fraction of Tregs being depleted by this drug, and that, should the Treg depletion and T cell activation be maintained longer, they might have resulted in mucosal damage, non-AIDS comorbidities, disease progression, and death. The incomplete Treg depletion is expected, because the drug does not directly target Tregs, but rather those cell subsets expressing the relatively nonspecific marker CD25. Note, however, that the impact on Treg function was significant, as demonstrated by the observation that we ablated the regulatory functions of the Tregs (54), with massive T cell activation following Ontak administration, and significantly altered the dynamics of key parameters of SIV infection. Meanwhile, a more profound Treg depletion might have been undesirable, as it could have induced autoimmunity, similar to the severe autoimmune and inflammatory disorders observed in human newborns without functional Tregs (55, 56) and in mice with near-complete Treg depletion (57, 58). Meanwhile, these mild effects of the partial Treg depletion played to our advantage, as they were not associated with gut damage, allowing us to dissociate T cell activation from inflammation.

Finally, it might be argued that Treg depletion had a minimal impact on the infiltration of natural killer cells into B cell follicles of the LNs and gut lymphoid tissue, a process proposed to be central to AGMs’ ability to clear the virus from the LN sanctuaries and preserve CD4^+^ T cell homeostasis (59, 60). However, while Treg depletion significantly activated CD8^+^ cells, irrespective of their phenotype, we did not observe a discernible effect on SIV infection outcome in the Ontak-treated AGMs, and thus NK activation likely did not impact the outcome of Ontak treatment.

Together, our results demonstrate that, in spite of the multifactorial nature of the persistent T cell immune activation and inflammation in chronic HIV/SIV infection, the key contributor to the control of chronic inflammation, comorbidities, and disease progression in the natural hosts of SIVs is the integrity of the mucosal barrier. The corollary of this observation is that, in pathogenic SIV infections, similar to HIV infection, mucosal immune dysfunction is the main determinant of persistent inflammation, which is strongly associated with comorbidities, progression to AIDS, and death. Our results thus point to the conclusion that focusing only on tackling T cell immune activation and systemic inflammation will likely not be effective for controlling HIV disease progression and comorbidities. Instead, our therapeutic interventions should target the causes of systemic inflammation and immune activation and hence attempt to restore gut integrity and reverse mucosal dysfunction in people living with HIV through specific therapies, dietary adjustment, probiotic/prebiotic administration (61–63), and even microbial transplantation (64).

## Methods

### Ethics statement.

Caribbean AGMs of both sexes were included in this study. All animals were housed and maintained at the Tulane National Primate Research Center of the Tulane University according to the standards of the Association for Assessment and Accreditation of Laboratory Animal Care. The animals were fed and housed according to regulations set forth by the *Guide for the Care and Use of Laboratory Animals* and the Animal Welfare Act (65). All AGMs included in this study were socially housed (paired) indoors in stainless steel cages, had 12-hour light/12 -hour dark cycle, were fed twice daily, and had water provided ad libitum. A variety of environmental enrichment strategies were employed, including housing of animals in pairs, providing toys to manipulate, and playing entertainment videos in the animal rooms. In addition, the animals were observed twice daily, and any signs of disease or discomfort were reported to the veterinary staff for evaluation. For sample collection, animals were anesthetized with 10 mg/kg ketamine HCl (Park-Davis) or 0.7 mg/kg tiletamine HCl and zolazepan (Telazol, Fort Dodge Animal Health) injected intramuscularly. At the completion of the study, the AGMs were sacrificed by intravenous administration of barbiturates.

### Animals, infection, treatment, and samples.

Nine adult AGMs of Caribbean origin (aged 4–7 years) were included. All animals were females. Four animals received 3 Ontak (Ligand Pharmaceutical) treatments (15 μg/kg) consisting of daily administration for 5 consecutive days each starting at –2, 32, and 53 dpi. Five AGMs served as controls: they were infected and were subjected to a similar follow-up, but they did not receive any treatment. All animals were infected by intravenous inoculation with plasma equivalent to 300 50% tissue culture-infective doses of the AGM’s SIVsab92018 (66). Animals were clinically monitored throughout the follow-up.

Blood was collected from all AGMs preinfection, to establish the baselines, at the initiation of Ontak, twice per week during the first 2 weeks, weekly up to 7 weeks postinfection, every 2 weeks up to 3 months, and then monthly until the end of the follow-up. An additional blood draw was performed on all the AGMs 2 days after each Ontak administration.

### LNs and intestinal biopsies.

LNs and intestinal biopsies were collected as described (38, 66, 67) preinfection, during the acute SIV infection, at the end of the acute infection, at the setpoint, and at 2 time points during the chronic infection. Additional LN and intestinal samples were collected during the necropsy.

### Viral quantification.

pVLs were quantified as previously described (66). Assay limit of quantification was 30 copies/mL. CA-vRNA was also quantified on cells isolated from the intestinal biopsies, as described (38).

### Isolation of PBMCs and of lymphocytes from LNs and intestinal biopsies.

PBMCs were purified from whole blood by density gradient centrifugation using lymphocyte separation medium (Organon-Technica). Lymphocytes were separated from LNs by pressing tissue through a nylon mesh screen. Cells were filtered through nylon bags and washed with RPMI medium (Cellgro) containing 5% heat-inactivated newborn calf serum, 0.01% penicillin-streptomycin, 0.01% l-glutamine, and 0.01% HEPES buffer, as previously described (38, 68). Intestinal biopsies were washed with EDTA, then subjected to collagenase digestion (MilliporeSigma). Cell suspension was filtered through a 70 μm filter (Thermo Fisher Scientific), then layered in a tube containing 2 mL of a 35% Percoll solution on top of 2 mL of a 60% Percoll solution. After a centrifugation at 1,023*g* for 20 minutes, lamina propria lymphocytes were retrieved at the interphase between the 2 Percoll solutions. LNs were mechanically minced, pressed through a 70 μm nylon mesh screen, filtered through 70 μm nylon mesh bags, and washed with RPMI medium containing 5% heat-inactivated newborn calf serum, 0.01% penicillin-streptomycin, 0.01% l-glutamine, and 0.01% HEPES buffer, as described (32, 38). Freshly isolated cells were then used for flow cytometry.

### Antibodies and flow cytometry.

Whole blood was stained for flow cytometry as previously described (38, 68). mAbs used were as follows: CD3- FITC (clone SP34) or CD3-PerCP (clone SP34-2); CD20-PE (clone 2H7); CD8-PerCP (clone SK1) or CD8-PE (clone RPA-T8); CD4-APC (clone L200) or CD4-PerCP (clone L200); HLA-DR-PerCP (clone L243); CD95-FITC (clone DX2) or CD95-APC (clone DX2); CD28-APC (clone CD28.2) or CD28-PE (clone CD28); Ki-67–FITC (clone B56); CD25-FITC (anti–IL-2 receptor) (clone 2A3); and FoxP3–Alexa Fluor 488 (clone 259D.C7) (all from BD Biosciences). All Abs were validated and titrated using AGM PBMCs. Data were acquired with an LSR II flow cytometer (BD Biosciences) and analyzed with CellQuest software (BD Biosciences). CD4^+^ and CD8^+^ T cell percentages were obtained by first gating on lymphocytes and then on CD3^+^ T cells. Memory, activation, and proliferation markers were determined by gating on lymphocytes, then on CD3^+^ T cells, and finally on CD4^+^CD3^+^ or CD8^+^CD3^+^ T cells. Gating strategy for Tregs is presented in Supplemental Figure 6, while the gating strategy for macrophages is presented in Supplemental Figure 7.

### IHC.

IHC was used for monitoring FoxP3 expression and multiple markers of gut integrity using paraffin-included intestinal samples collected at the necropsy from both Ontak-treated and control AGMs, as previously reported (46). Additional intestinal samples from uninfected AGMs from other studies (27) were used as negative controls. IHC was performed on formalin-fixed, paraffin-embedded LN samples. Four μm–thick sections were deparaffinized, rehydrated, and rinsed in distilled H_2_O. For antigen retrieval, sections were microwaved in Vector Unmasking Solution (Vector Laboratories) and treated with 3% hydrogen peroxide to quench endogenous peroxidases. Slides were incubated with Dako Protein Blocking Serum for 30 minutes, followed by a 1-hour incubation with the primary antibody (Supplemental Table 1). Secondary antibody (Supplemental Table 1) and actin/biotin (Vector Vectastain ABC Kit, Vector Laboratories) incubations were performed consecutively thereafter. For visualization, slides were stained with diaminobenzidine (Dako) and counterstained with hematoxylin. Slide images were quantified using FIJI image software, using 5 images per section, per time point, per animal. Regions of interest (ROIs) within each slide were identified, and positive signals within the ROIs were selected via color threshold and measured/averaged by percentage area positive, as previously described (46).

For a more accurate representation of the impact of Ontak treatment on the mucosal epithelium, we quantified Ki-67 and claudin-3 through more specific methods. For Ki-67, we measured the length of the crypts with Ki-67^+^ epithelial cells versus the total length of the crypts (Supplemental Figure 5). This was done by manually drawing a black line through the middle of the crypts from the submucosal ends to the luminal ends, then bifurcating the line based on the relative position of the Ki-67^+^ epithelial cells and measuring the 2 line segments with the FIJI Wand tool, as previously described (14). This allowed us to quantify the fraction of proliferating and activated mucosal epithelial cells to establish a ratio between the two.

For claudin-3, multiple overlapping images of the gut epithelium were acquired and used to create a composite image using the Image Stitching plug-in (69) in the FIJI software (70, 71). Once the composite image was generated, we first traced the length of the intact epithelium with a green line and then traced any broken or damaged sections with a red line (Supplemental Figure 5A). The total lengths of the 2 lines were then measured using the FIJI Wand tool to establish the length of intact versus broken epithelium, as previously described (14).

The antibody clones used for IHC, as well as their origin, are listed in Supplemental Table 1.

### Cytokine and chemokine testing.

Cytokine testing in plasma was done using a sandwich immunoassay–based protein array system, the Cytokine Monkey Magnetic 28-Plex Panel (Invitrogen), as described (32, 72) and according to the manufacturer’s instructions. Results were read using the Bio-Plex array reader (Bio-Rad Laboratories), which uses fluorescent bead–based technology (Luminex Corporation).

### CRP testing.

CRP is an acute-phase protein that rises in the plasma in response to inflammation. It binds to the phosphocholine expressed on the surface of dead cells and some types of bacteria to activate the complement via the C1Q complex. CRP is a biomarker associated with death in HIV-infected patients (7). CRP was measured using a monkey CRP ELISA kit (Life Diagnostics).

### Assessment of the levels of microbial translocation.

Plasma levels of LPS were measured as described (13, 15). Multiple plasma factors can interfere with LPS measurements (LBP, EndoCAb, HDL, plasma turbidity, proteins, and triglycerides). Therefore, to minimize any possible interference, plasma samples were diluted 5-fold with endotoxin-free water, then heated to 85°C for 15 minutes to inactivate plasma proteins. Plasma LPS was quantified with a commercially available *Limulus* amebocyte lysate assay (Cambrex), according to manufacturer’s protocol. Each sample was run in duplicate.

Plasma sCD163 levels were also measured as a surrogate marker of microbial translocation (13, 15). sCD163 is a soluble marker of macrophage activation, which is shed to serum by inflammatory signals, including LPS. The circulating levels of sCD163 are a surrogate for direct measurement of endotoxin or Gram-negative bacteria translocated from the intestinal lumen to circulation as a result of the immunologic injury inflicted at the mucosal level by pathogenic HIV/SIV infection (9, 13). Plasma sCD163 levels were measured by ELISA (Trillium Diagnostics, LLC); analytical coefficient of variation ranged from 4.6% to 13.6%.

### I-FABP.

The plasma I-FABP level was quantified with a monkey I-FABP ELISA kit (MyBioSource, Inc.), as described (46).

Neopterin is produced by human monocytes/macrophages upon cytokine stimulation. Therefore, measurement of neopterin concentrations in body fluids like serum, cerebrospinal fluid, or urine provides information about macrophage activation. Plasma neopterin levels were measured using an ELISA kit (Thermo Fisher Scientific) as per manufacturer’s instructions.

### Coagulation status.

Coagulation status was estimated by determining plasma levels of D-dimer (DD). DD is a fibrin degradation product present in the blood after a blood clot is degraded by fibrinolysis. DD independently correlates with lentiviral disease progression and death in HIV-infected patients (7) and SIV-infected macaques (9). DD was measured using a STAR automated coagulation analyzer (Diagnostica Stago) and an immunoturbidimetric assay (Liatest D-DI, Diagnostica Stago). We previously optimized this assay for use in AGMs (9).

### Endothelial activation.

Activation was assessed by measuring the plasma levels of P-selectin. P-selectin is a cell adhesion molecule on the surfaces of activated endothelial cells and platelets and acts as a receptor that supports binding of leukocytes to activated platelets and endothelium. Plasma P-selectin levels are markedly elevated in inflammation and thrombogenic conditions (73). The plasma-soluble P-selectin levels were measured with monkey sP-selectin Platinum ELISA (eBioscience), as per the manufacturer’s instructions.

### Statistics.

Comparison of single postinfection time point value with preinfection, for each parameter, was done using the Wilcoxon signed-rank test. For the majority of analyses comparing treated macaques with controls, we used linear mixed effects models (74), where the parameter of interest was the dependent variable, time and treatment were the independent variables, and macaque was the grouping factor. We compared the effect of treatment of the dependent variable in 3 time periods separately: acute phase (up to 21 dpi), postacute-to-chronic transition (from 21 dpi to 80 dpi), and late chronic (≥80 dpi), as the effect of the last Ontak administration at 53 dpi starts to wear off. In the linear mixed effects approach, we used all the measurements available together and used macaque as the grouping (or random) factor to account for the repeated measurements made in each animal. For variables in the gut, where fewer measurements were available, we were not able to assess the individual time periods separately and evaluated differences throughout the follow-up to 80 dpi. Whenever possible, we tested multiple models with fixed effects for time and treatment, with or without interactions. In this way, we were analyzing not only differences in the levels of the variable between treated and control monkeys but also whether there is a difference in the variability of those levels over time (corresponding to the interaction term). The best model for the data (with or without the interaction term) was chosen by comparing the log likelihood. For these analyses we used the lme function of the nlme package of R (http://cran.r-project.org/). We evaluated correlations between markers of interest using generalized estimating equations (gee) using an exchangeable working correlation structure. For these analyses, we used the geepack package of R. Any *P* value less than 0.05 was considered significant.

### Study approval.

Experiments described in this study were approved by the Tulane University Institutional Animal Care and Use Committee (IACUC protocol 3409, approved in 2008).

### Data availability.

All the data used in the paper are available from the corresponding authors upon request.

## Author contributions

CA and IP designed the research studies; CA, EBC, TH, KJM, AJK, PS, RS, SS, NK, KDR, TG, NMS, RG, AV, and IP conducted the experiments; EBC, TG, KJM, RS, PS, TG, AV, and RG acquired data; CA, AAL, RMR, ALL, and IP analyzed data; and CA and IP wrote the manuscript.

## Supplementary Material

Supplemental data

## Version 1 07/24/2023

Electronic publication
