# Supplementary material for: T cell activation is insufficient to drive SIV disease progression
Source: JCI Insight. 2023 Jul 24;8(14):e161111. doi: 10.1172/jci.insight.161111 (PMC10443804; doi:10.1172/jci.insight.161111)
Supplement: Supplemental data [file jciinsight-8-161111-s073.pdf]

T-Cell Activation is Insufficient to Drive SIV Disease Progression

----

Cristian Apetrei,[1,3,4*] Thaidra Gaufin,[4] Egidio Brocca-Cofano, Ranjit Sivanandham,[2] Paola Sette,[1] Tianyu He,[2] Sindhuja Sivanandham,[2] Natalie Martinez Sosa,[1] Kathryn J. Martin,[1] Kevin D. Raehtz,[1] Adam J. Kleinman,[1] Audrey Valentine,[2] Noah Krampe,[2] Rajeev Gautam,[4] Andrew A. Lackner,[4] Alan L Landay,[5] Ruy M. Ribeiro,[6,7] and Ivona Pandrea[2,3,4*]

SUPPLEMENTARY INFORMATION

Ontak administration did not hinder the SIV-specific cellular immune responses. In the past, studies have reported that IL-2-diphteria toxin fusion protein can abolish cell-mediated immunity (1). To assess whether or not the effects on SIV pathogenesis observed after Ontak administration are due to the therapeutic effects of the drug or to an impairment of cell mediated immune responses, we next monitored the dynamics of SIV-specific T cells in AGMs receiving Ontak.

Functional activity of both $CD4^+$ and $CD8^+$ T cells were monitored by intracellular cytokine staining (ICS) measurements of IL-2, TNF-α, IFN-γ, CD107α and MIP-1β production in response to stimulation with SIVsab92018 Gag or Env peptide pools, measured in samples obtained throughout the follow-up, before, and after Ontak administration. Our focus was on the CD107α, which is considered a correlate of the cytotoxic potential of SIV-specific T cells. This experiment did not find a significant loss of the Gag and Env-specific $CD4^+$ and $CD8^+$ T cells (Supplemental Figures S2 and S3). On the contrary, Ontak administration slightly boosted the Gag and Env-specific $CD4^+$ and $CD8^+$ T cells (Supplemental Figures S2 and S3), albeit these increases did not

reach statistical significance. Furthermore, polyfunctionality of the $CD4^+$ and $CD8^+$ T cells was maintained or even boosted after Ontak administration. A sizable proportion of the SIV-specific T cells were positive for the degranulation marker CD107α. Furthermore, the frequency of SIV-specific CD107α positive cells increased after Ontak administration. The same pattern was observed after every Ontak administration. These results suggest that Ontak boosted the SIV-specific T cells *in vivo*, probably as a result of the Treg depletion.

Methods

To assess the dynamics of SIV-specific T lymphocytes after Ontak administration, functional assays were performed on frozen PBMCs after short stimulation with 15-mer peptide pools spanning the sequences of major antigenic proteins of SIVsab92018, in presence of anti-CD28 and anti-CD49d and brefeldin/monensin, as described (2, 3). PBMCs were stained with the following antibodies: CD3-V500 (SP34-2), CD4-APC (L200)), CD8-PE-CF594 (RPA-T8), CD107a-APC-Cy7 (H4A3), Granzyme A-PerCP/Cy5.5 (CB9) (BioLegend), Granzyme B-Alexa Flour 700 (GB11), Granzyme K-FITC (GM6C3) (Santa Cruz Biotechnology, Dallas, TX), IFN-γ-FITC (4S.B3), and Perforin-PE (B-D48) (BioLegend); antibodies were from BD Biosciences unless otherwise noted. Stained PBMCs were then acquired on an LSR-II flow cytometer. Data were analyzed using FlowJo software (Treestar), as described (2, 3).

Supplemental Table S1. **Primary and secondary antibodies for immunohistochemistry**

Primary Antibody	Description (Clone)	Dilution	Secondary antibody	Company
Anti-FoxP3	Mouse Monoclonal (Clone 259D)	1:100	Goat, anti-mouse	Biolegend, San Diego, CA,
Anti-MX1	Rabbit Polyclonal (ab95926)	1:2000	Goat, anti-rabbit	Abcam, Cambridge, MA
Anti-Claudin-3	Rabbit Polyclonal (RB-9251-P1)	1:100	Goat, anti-rabbit	ThermoScientific, Fremont, CA
Anti-Ki-67	Mouse Clone MIB-1 (cat:M7240)	1:100	Horse, anti-mouse	Dako, Carpinteria, CA
Anti-LPS	Mouse Clone WN1222-5 (cat: HM6011)	1:100	Horse, anti-mouse	Hycult Biotech, North Brabant, The Netherlands

Supplemental Figure Legends

Supplemental Figure S1. Ontak administration to SIVsab-infected AGMs depletes Tregs. (a) Comparison between $CD25^{high}$ $CD4^+$ T cell frequency dynamics in acutely SIVsab-infected AGMs receiving Ontak (red) and untreated controls (grey). (b) Comparison between $FoxP3^+$ $CD4^+$ T cell frequency dynamics in acutely SIVsab-infected AGMs receiving Ontak (red) and untreated controls (grey). Shown are both the individual levels in treated *vs* untreated AGMs (a and c) and the average values for each group and standard error of means (b and d). Time points of Ontak treatment initiation (-2, 32 and 53 dpi) are marked by red arrows.

Supplemental Figure S2. Ontak administration boosts the overall SIV-specific T cell responses in SIVsab-infected AGMs. Absolute numbers (/ml) of the aggregated Gag- and Env-specific $CD4^+$ and $CD8^+$ T cells, as determined by intracellular cytokine staining and flow cytometry, are shown at different time points following different rounds of Ontak administration. On the X axis, the numbers represent days post-SIVsab infection. Ontak administration time points are illustrated as red arrows.

Supplemental Figure S3. Representative results of the SIV-specific T cell responses at different time points postinfection and post-ONTAK administration for AGM T1. PBMCs from the AGMs were stimulated with peptides pools of Env (peptides 1-52) and stained for IFN-γ (I), MIP-1β (M), IL-2 (2), TNF-α (T), and CD107a (LAMP-1). Total percentages of $CD4^+$ T cells producing effector molecules are presented inside of the circles, while color coding of the rings indicate whether 1, 2, 3, 4, or 5 effector molecules are being produced. Shown are representative results for testing

SIV-specific immune responses for $CD4^+$ T cells: Env peptide pool 1-52 (A); Gag1 peptide pool 1-68 (B) and Gag2 peptide pool 69-136 (C), as well as for $CD8^+$ T cells: Env peptide pool 1-52 (D); Gag1 peptide pool 1-68 (E) and Gag2 peptide pool 69-136 (F).

Supplemental Figure S4. Ontak administration to SIVsab-infected AGMs increases the frequency of recently migrated macrophages (Mφ) to the lymph nodes, but the frequency of the activated macrophages decreases proportionally, such as the overall activation status does not significantly changes. Shown are both the individual levels in treated *vs* untreated AGMs for (A) total Mφ ($CD14^+$), (B) in the LNs, as well as (B) the overall Mφ activation status ($CD80^+$ $CD86^+$). Ontak-treated SIV-infected AGMs are depicted in red. Controls are depicted in grey. Time points of Ontak treatment initiation (-2, 32 and 53 dpi) are shown by red arrows.Statistical differences between the two groups are illustrated by asterisks (*).

Supplemental Figure S5. A. Tracing of continuous gut epithelium to establish ratio of intact *vs*. damaged epithelium in SIV-infected AGMs. A large composite image was constructed out of multiple smaller images to allow for better epithelium tracing. The longest stretch of intact epithelium was then selected and traced by hand with a line of fixed width. Intact sections were traced with green and damaged sections were traced in red. Then, the total number of pixels in the intact and damaged sections were measured and added to obtain the total length of the line, which was then used to calculate the ratio of intact/damaged epithelium *vs*. total epithelium. The gut section shown here was sampled from the jejunum of a chronically SIV-infected AGM that received Ontak and is displayed before (left) and after (right) manual tracing. All images were captured at 100X magnification using an AxioImager M1 brightfield microscope equipped with an AxioCam MRc5. All image measurements were done with

FIJI v.1.0. **B. Measurement of the length of Ki-67$^+$ epithelial cells *vs* the total length of the crypt.** Epithelial proliferation being localized to the base of the crypts, to establish a ratio of proliferating versus nonproliferating epithelial cells, we measured both the entire length of the crypt (total black line) and the length of the Ki-67$^+$ epithelial cells (black line below the bisecting pink arrow). Length was normalized by the number of pixels in each line segment All lines and arrows were drawn at a fixed width. Image from an uninfected AGM and is displayed before (left) and after (right) manual tracing. All individual images were captured at 200X magnification using an AxioImager M1 brightfield microscope equipped with an AxioCam MRc5. Image measurements were done with FIJI v.1.0.

Supplemental Figure S6. Gating strategy employed to characterize CD4$^+$ T cells expressing either CD25 or FoxP3 (regulatory T cells-Tregs. CD4$^+$ and CD8$^+$ T cells were gated on singlets followed by lymphocytes and CD3$^+$; CD4$^+$ T cells expressing CD25 (upper panels) or FoxP3 (lower panels) were next selected. Expression of both CD25and FoxP3 are shown before and after Ontak administration.

Supplemental Figure S7. Gating strategy employed to characterize monocytes/macrophages and their activation status. On the monocyte cloud, lineage negative cells were first selected, then, from this population was selected the fraction of cells expressing HLA-DR. From this population, the fraction expressing CD14 was selected.

Supplemental References

1. Kelley VE, Bacha P, Pankewycz O, Nichols JC, Murphy JR, and Strom TB. Interleukin 2-diphtheria toxin fusion protein can abolish cell-mediated immunity in vivo. *Proceedings of the National Academy of Sciences of the United States of America.* 1988;85(11):3980-4.
2. He T, Brocca-Cofano E, Policicchio BB, Sivanandham R, Gautam R, Raehtz KD, et al. Cutting Edge: T regulatory cell depletion reactivates latent simian immunodeficiency virus (SIV) in controller macaques while boosting SIV-specific T lymphocytes. *J Immunol.* 2016;197(12):4535-9.
3. Policicchio BB, Xu C, Brocca-Cofano E, Raehtz KD, He T, Ma D, et al. Multi-dose Romidepsin reactivates replication competent SIV in post-antiretroviral rhesus macaque controllers. *PLoS pathogens.* 2016;12(9):e1005879.

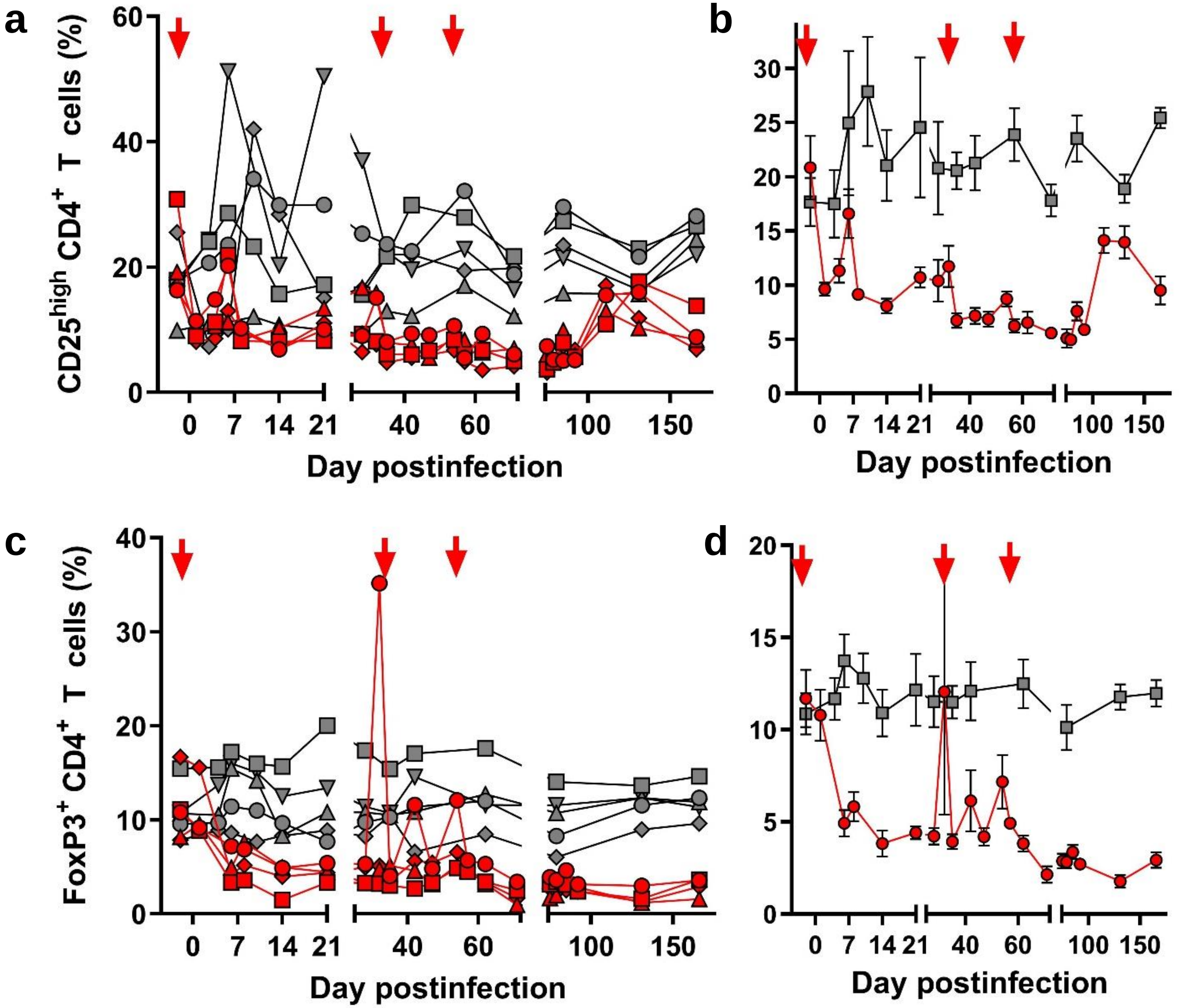

Supplemental Figure S1. Ontak administration to SIVsab-infected AGMs depletes Tregs. (**a**) Comparison between CD25^{high} CD4^{+} T cell frequency dynamics in acutely SIVsab-infected AGMs receiving Ontak (red) and untreated controls (grey). (b) Comparison between FoxP3^{+} CD4^{+} T cell frequency dynamics in acutely SIVsab-infected AGMs receiving Ontak (red) and untreated controls (grey). Shown are both the individual levels in treated *vs* untreated AGMs (a and c) and the average values for each group and standard error of means (b and d). Time points of Ontak treatment initiation (-2, 32 and 53 dpi) are marked by red arrows

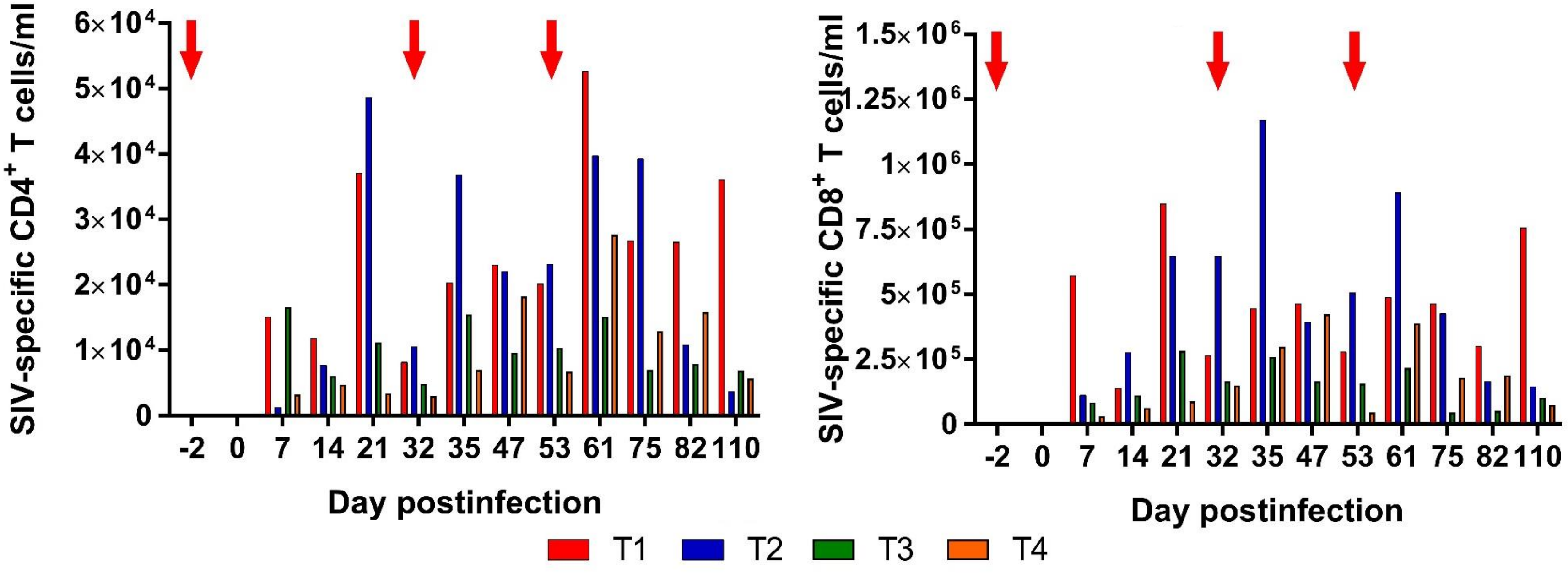

Supplemental Figure S2. Ontak administration boosts the overall SIV-specific T cell responses in SIVsab-infected AGMs. Absolute numbers (/ml) of the aggregated Gag- and Env-specific $CD4^+$ and $CD8^+$ T cells, as determined by intracellular cytokine staining and flow cytometry, are shown at different time points following different rounds of Ontak administration. On the X axis, the numbers represent days post-SIVsab infection. Ontak administration time points are illustrated as red arrows.

A. AGM T1: SIV-specific CD4$^+$ T cells (anti-ENV pool peptides 1-52)

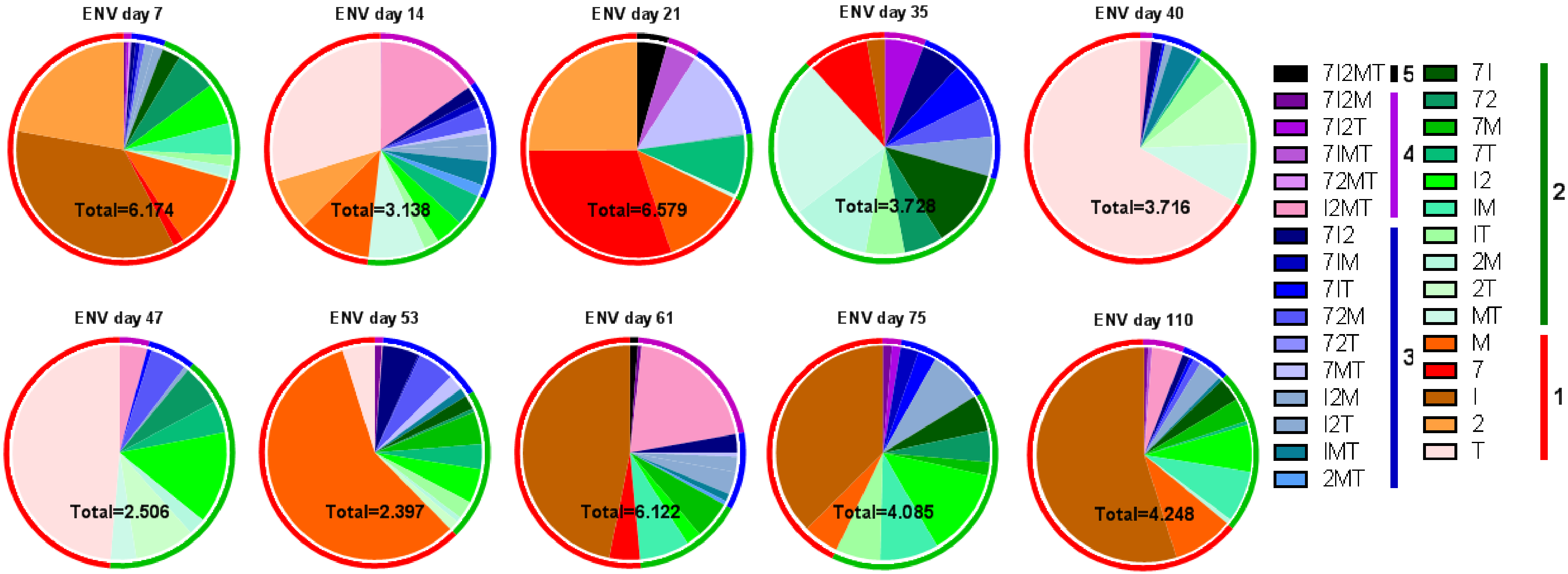

Supplemental Figure S3 A. Representative results of the SIV-specific (Env peptide pool 1-52) CD4$^+$ T cell responses at different time points postinfection and post-ONTAK administration for AGM T1. PBMCs from the AGMs were stimulated with peptides pools of Env (peptides 1-52) and stained for IFN-γ (I), MIP-1β (M), IL-2 (2), TNF-α (T), and CD107a (LAMP-1). Total percentages of CD4$^+$ T cells producing effector molecules are presented inside of the circles, while color coding of the rings indicate whether 1, 2, 3, 4, or 5 effector molecules are being produced.

B. AGM T1: SIV-specific CD4$^+$ T cells (anti-GAG1 pool peptides 1-68)

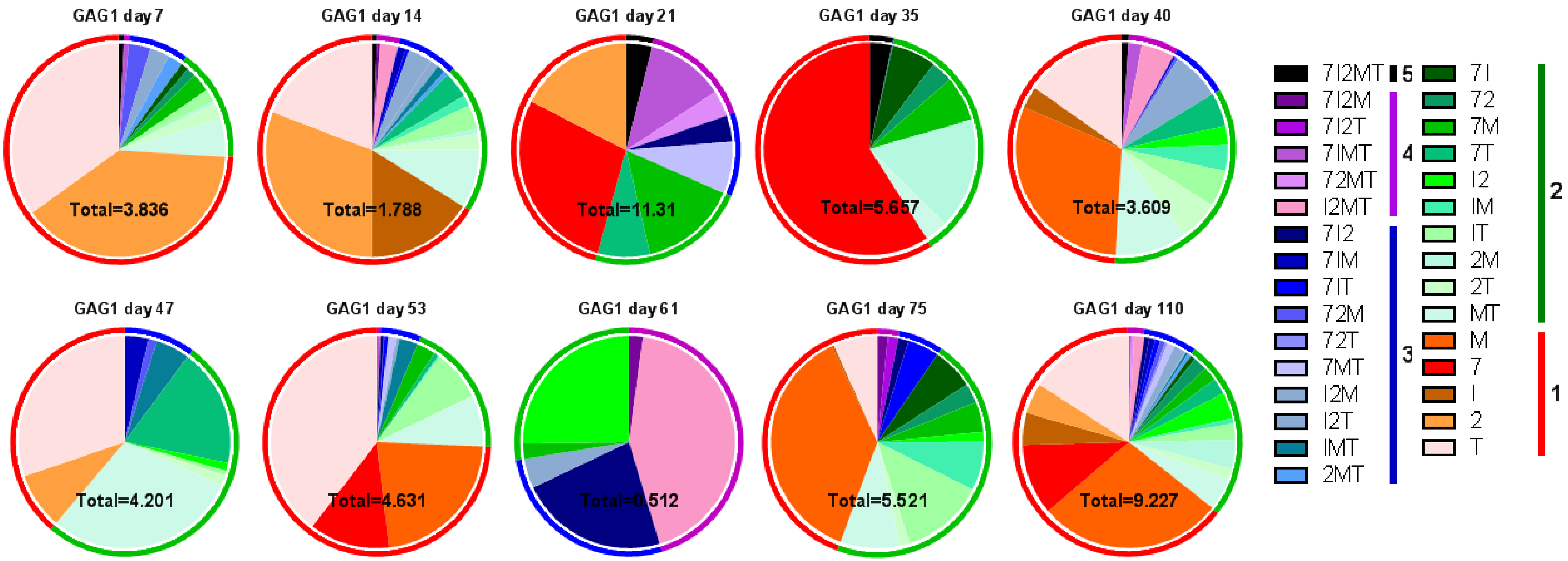

Supplemental Figure S3 B. Representative results of the SIV-specific (Gag1 peptide pool 1-68) CD4$^+$ T cell responses at different time points postinfection and post-ONTAK administration for AGM T1. PBMCs from the AGMs were stimulated with peptides pools of Gag1 (peptides 1-68) and stained for IFN-γ (I), MIP-1β (M), IL-2 (2), TNF-α (T), and CD107a (LAMP-1). Total percentages of CD4$^+$ T cells producing effector molecules are presented inside of the circles, while color coding of the rings indicate whether 1, 2, 3, 4, or 5 effector molecules are being produced.

C. AGM T1: SIV-specific CD4⁺ T cells (anti-GAG2 pool peptides 69-136)

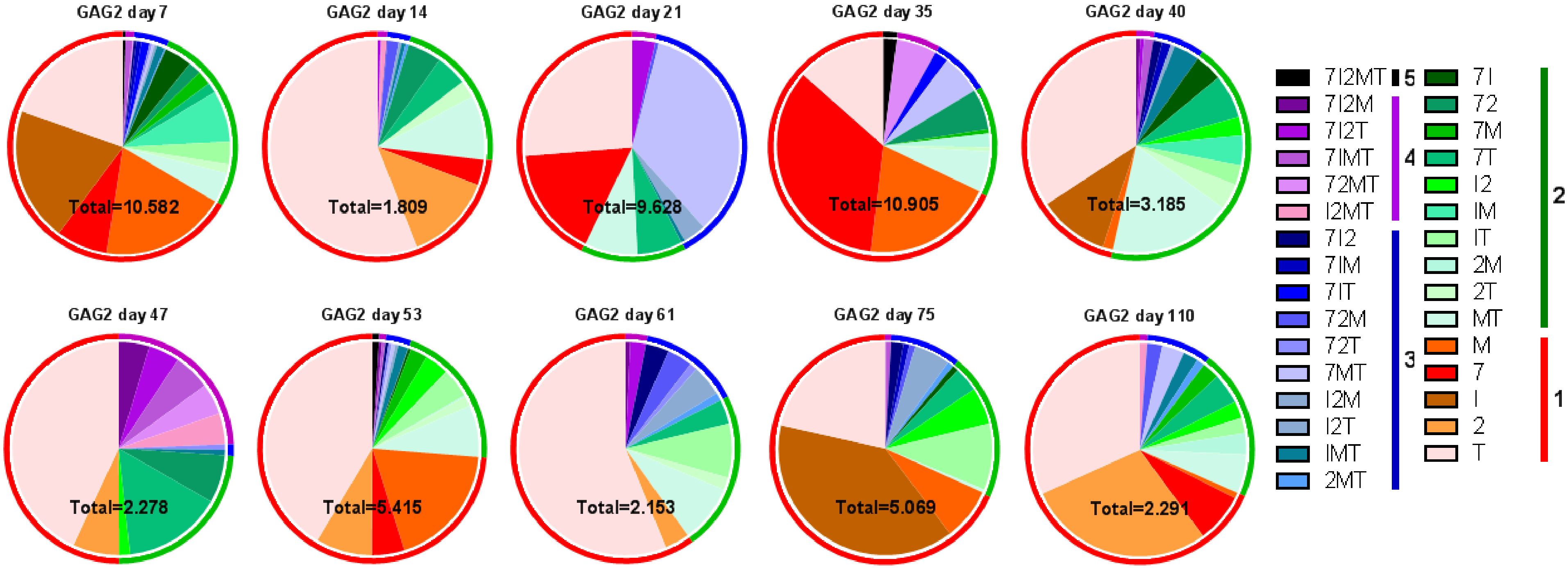

Supplemental Figure S3 C. Representative results of the SIV-specific (Gag2 peptide pool 69-136) CD4⁺ T cell responses at different time points postinfection and post-ONTAK administration for AGM T1. PBMCs from the AGMs were stimulated with peptides pools of Gag2 (peptides 69-136) and stained for IFN-γ (I), MIP-1β (M), IL-2 (2), TNF-α (T), and CD107a (LAMP-1). Total percentages of CD4⁺ T cells producing effector molecules are presented inside of the circles, while color coding of the rings indicate whether 1, 2, 3, 4, or 5 effector molecules are being produced.

D. AGM T1: SIV-specific CD8$^+$ T cells (anti-ENV pool peptides 1-52)

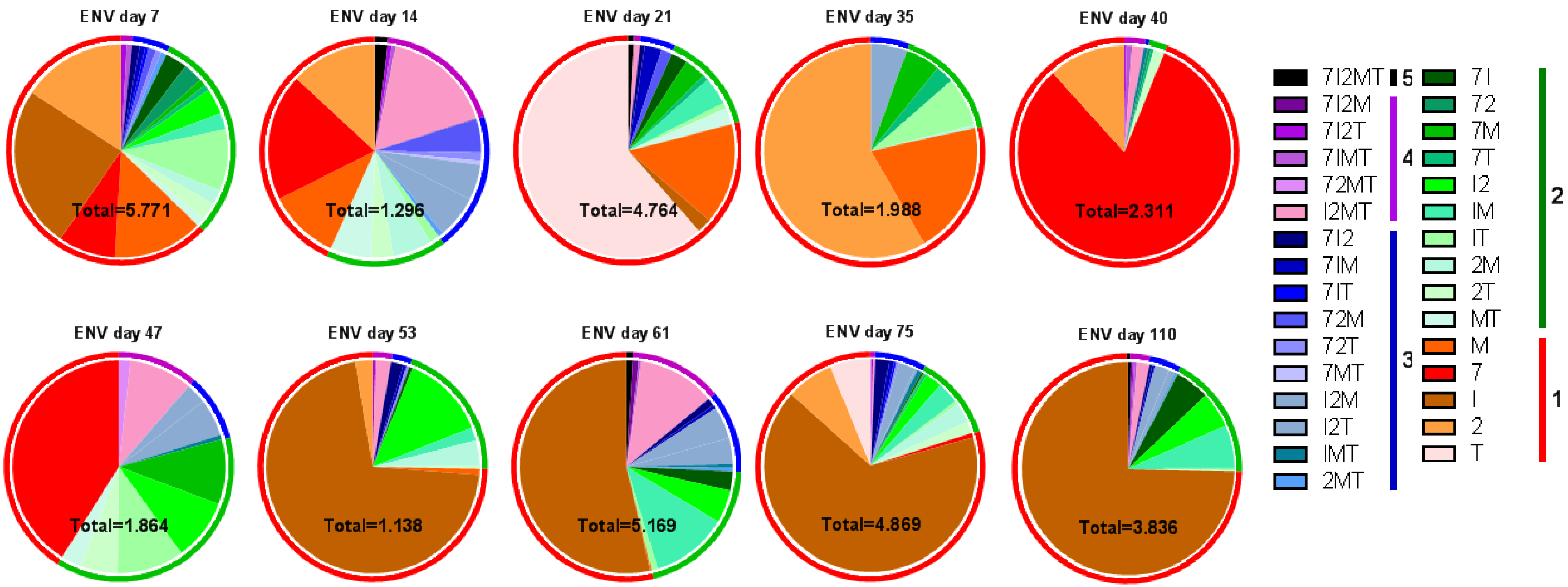

Supplemental Figure S3 D. Representative results of the SIV-specific (Env peptide pool 1-52) CD8$^+$ T cell responses at different time points postinfection and post-ONTAK administration for AGM T1. PBMCs from the AGMs were stimulated with peptides pools of Env (peptides 1-52) and stained for IFN-γ (I), MIP-1β (M), IL-2 (2), TNF-α (T), and CD107a (LAMP-1). Total percentages of CD8$^+$ T cells producing effector molecules are presented inside of the circles, while color coding of the rings indicate whether 1, 2, 3, 4, or 5 effector molecules are being produced.

E. AGM T1: SIV-specific CD8+ T cells (anti-GAG1 pool peptides 1-68)

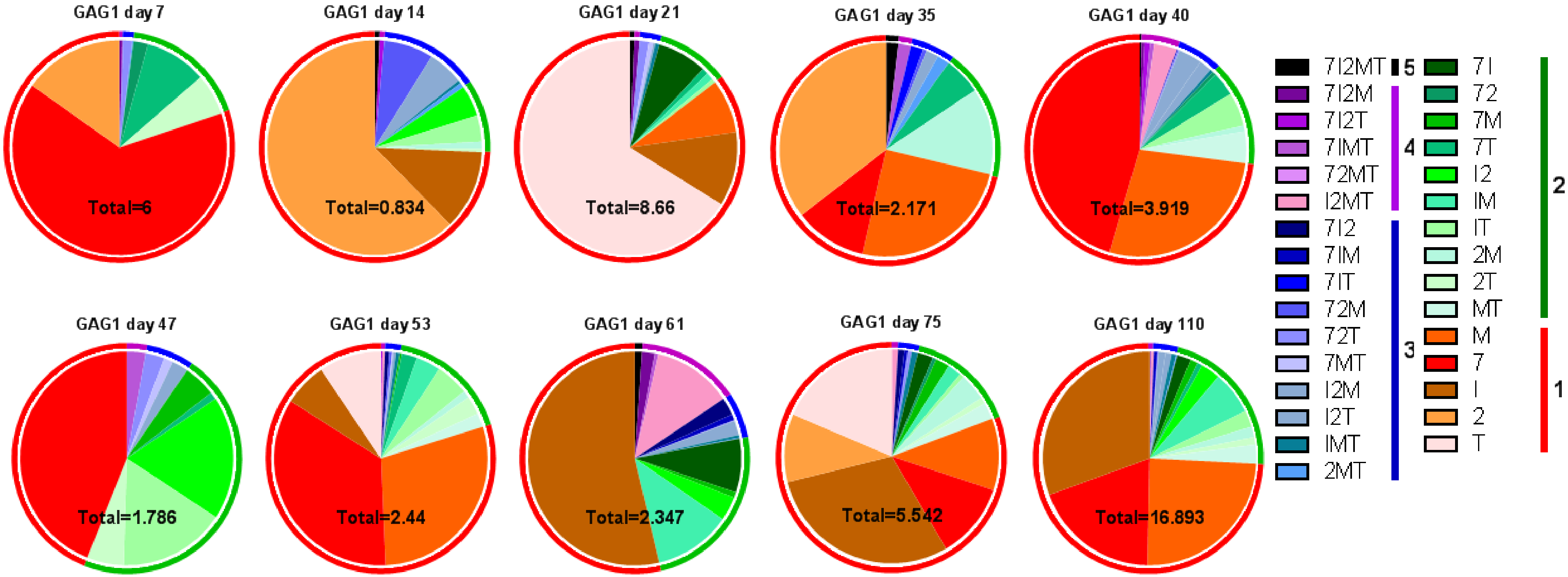

Supplemental Figure S3 E. Representative results of the SIV-specific (Gag1 peptide pool 1-68) CD8+ T cell responses at different time points postinfection and post-ONTAK administration for AGM T1. PBMCs from the AGMs were stimulated with peptides pools of Gag1 (peptides 1-68) and stained for IFN-γ (I), MIP-1β (M), IL-2 (2), TNF-α (T), and CD107a (LAMP-1). Total percentages of CD8+ T cells producing effector molecules are presented inside of the circles, while color coding of the rings indicate whether 1, 2, 3, 4, or 5 effector molecules are being produced.

F. AGM T1: SIV-specific CD8$^+$ T cells (anti-GAG2 pool peptides 69-136)

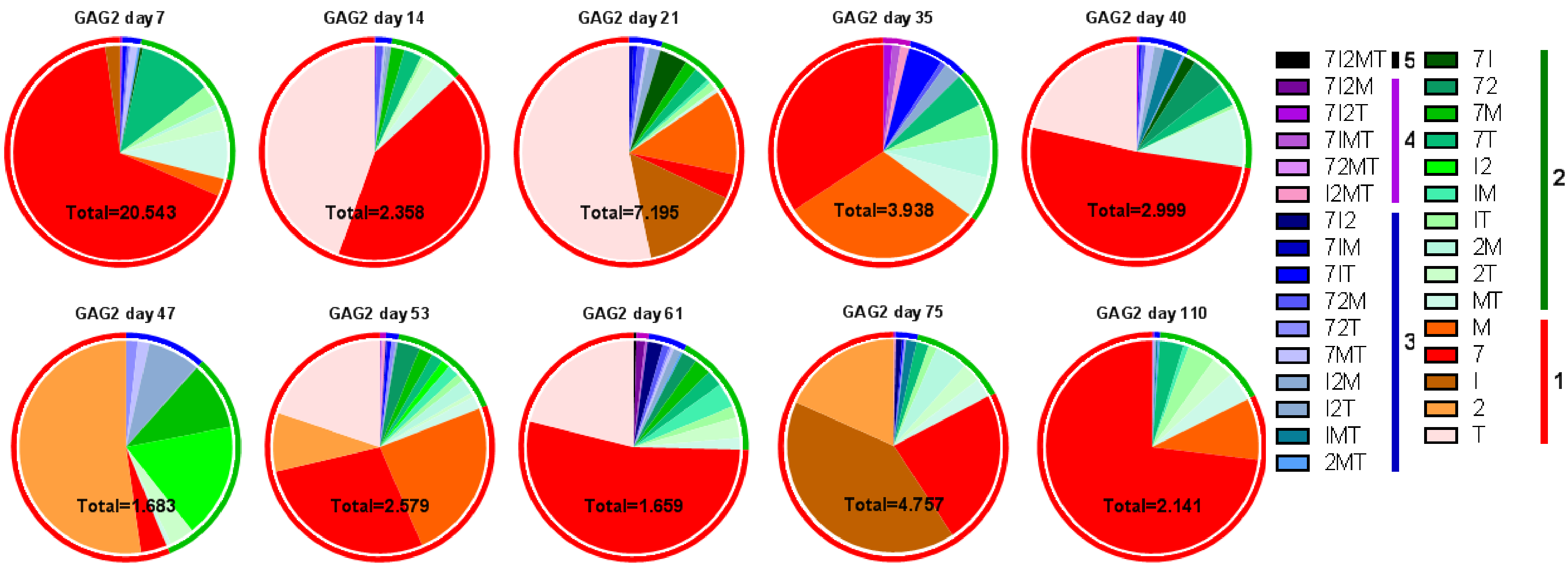

Supplemental Figure S3 F. Representative results of the SIV-specific (Gag2 peptide pool 69-136) CD8$^+$ T cell responses at different time points postinfection and post-ONTAK administration for AGM T1. PBMCs from the AGMs were stimulated with peptides pools of Gag2 (peptides 69-136) and stained for IFN-γ (I), MIP-1β (M), IL-2 (2), TNF-α (T), and CD107a (LAMP-1). Total percentages of CD8$^+$ T cells producing effector molecules are presented inside of the circles, while color coding of the rings indicate whether 1, 2, 3, 4, or 5 effector molecules are being produced.

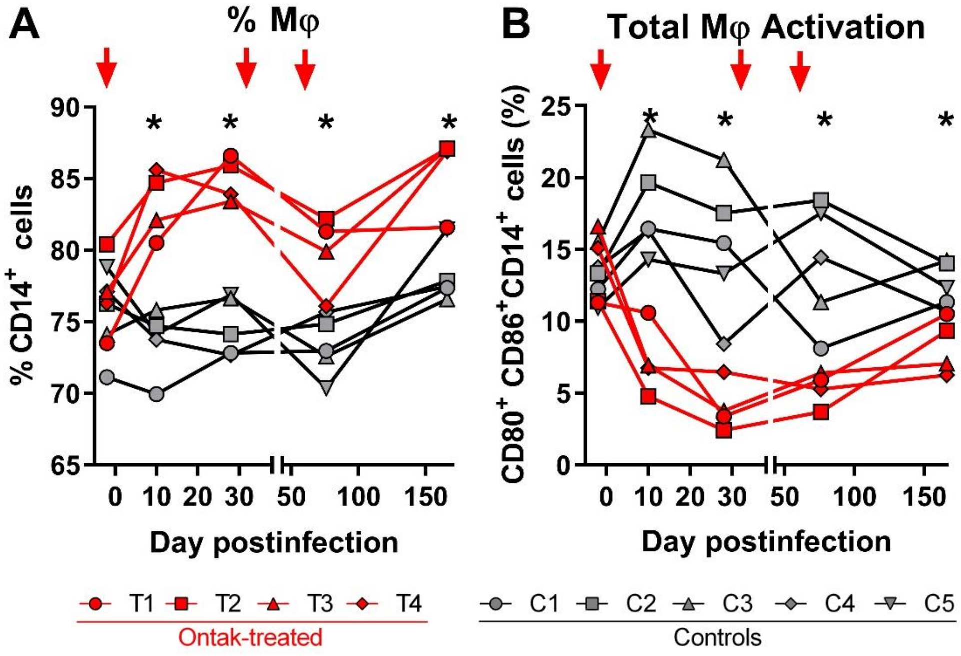

Suplemental Figure S4. Ontak administration to SIVsab-infected AGMs increases the frequency of recently migrated macrophages (Mφ) to the lymph nodes, but the frequency of the activated macrophages decreases proportionally, such as the overall activation status does not significantly changes. Shown are both the individual levels in treated *vs* untreated AGMs for (A) total Mφ ($CD14^+$), (B) in the LNs, as well as (B) the overall Mφ activation status ($CD80^+$ $CD86^+$). Ontak-treated SIV-infected AGMs are depicted in red. Controls are depicted in grey. Time points of Ontak treatment initiation (-2, 32 and 53 dpi) are shown by red arrows.Statistical differences between the two groups are illustrated by asterisks (*).

A

B

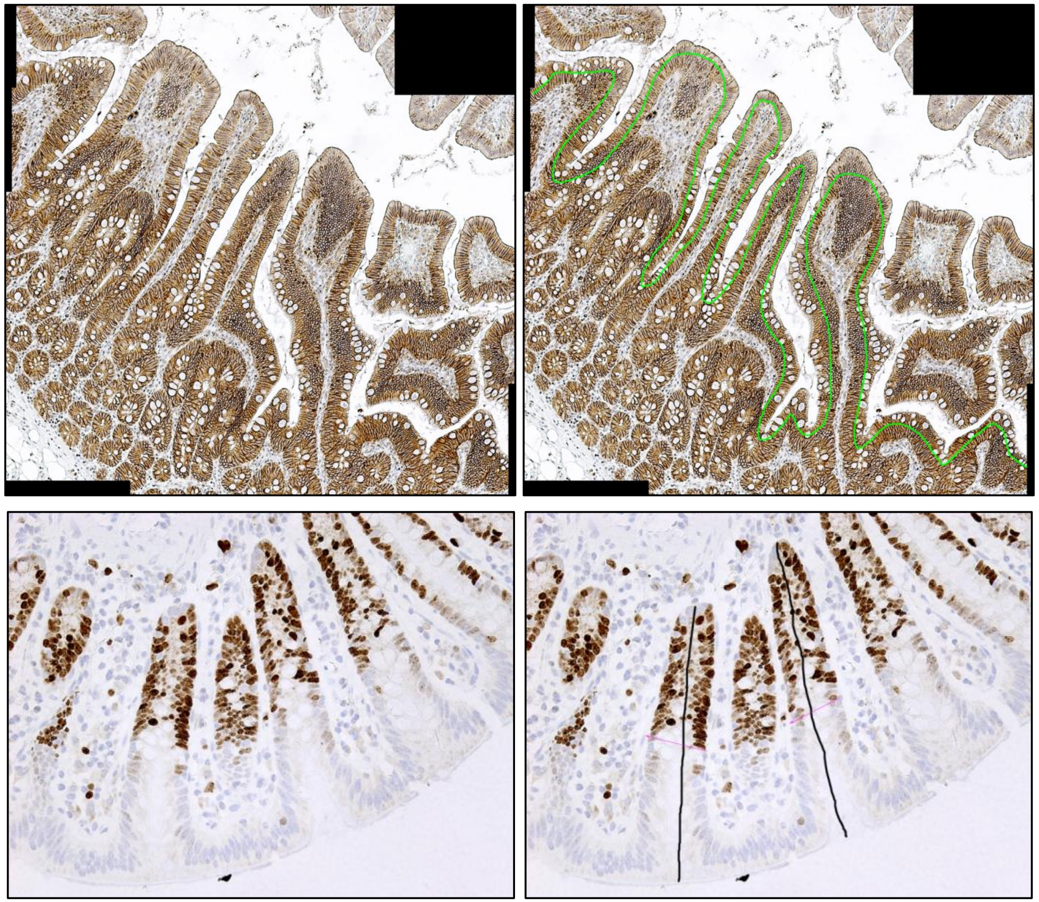

Supplemental Figure S5. A. Tracing of continuous gut epithelium to establish ratio of intact *vs*. damaged epithelium in SIV-infected AGMs. A large composite image was constructed out of multiple smaller images to allow for better epithelium tracing. The longest stretch of intact epithelium was then selected and traced by hand with a line of fixed width. Intact sections were traced with green and damaged sections were traced in red. Then, the total number of pixels in the intact and damaged sections were measured and added to obtain the total length of the line, which was then used to calculate the ratio of intact/damaged epithelium *vs*. total epithelium. The gut section shown here was sampled from the jejunum of a chronically SIV-infected AGM that received Ontak and is displayed before (left) and after (right) manual tracing. All images were captured at 100X magnification using an AxioImager M1 brightfield microscope equipped with an AxioCam MRc5. All image measurements were done with FIJI v.1.0. **B. Measurement of the length of Ki-67$^+$ epithelial cells *vs* the total length of the crypt.** Epithelial proliferation being localized to the base of the crypts, to establish a ratio of proliferating versus nonproliferating epithelial cells, we measured both the entire length of the crypt (total black line) and the length of the Ki-67$^+$ epithelial cells (black line below the bisecting pink arrow). Length was normalized by the number of pixels in each line segment All lines and arrows were drawn at a fixed width. Image from an uninfected AGM and is displayed before (left) and after (right) manual tracing. All individual images were captured at 200X magnification using an AxioImager M1 brightfield microscope equipped with an AxioCam MRc5. Image measurements were done with FIJI v.1.0.

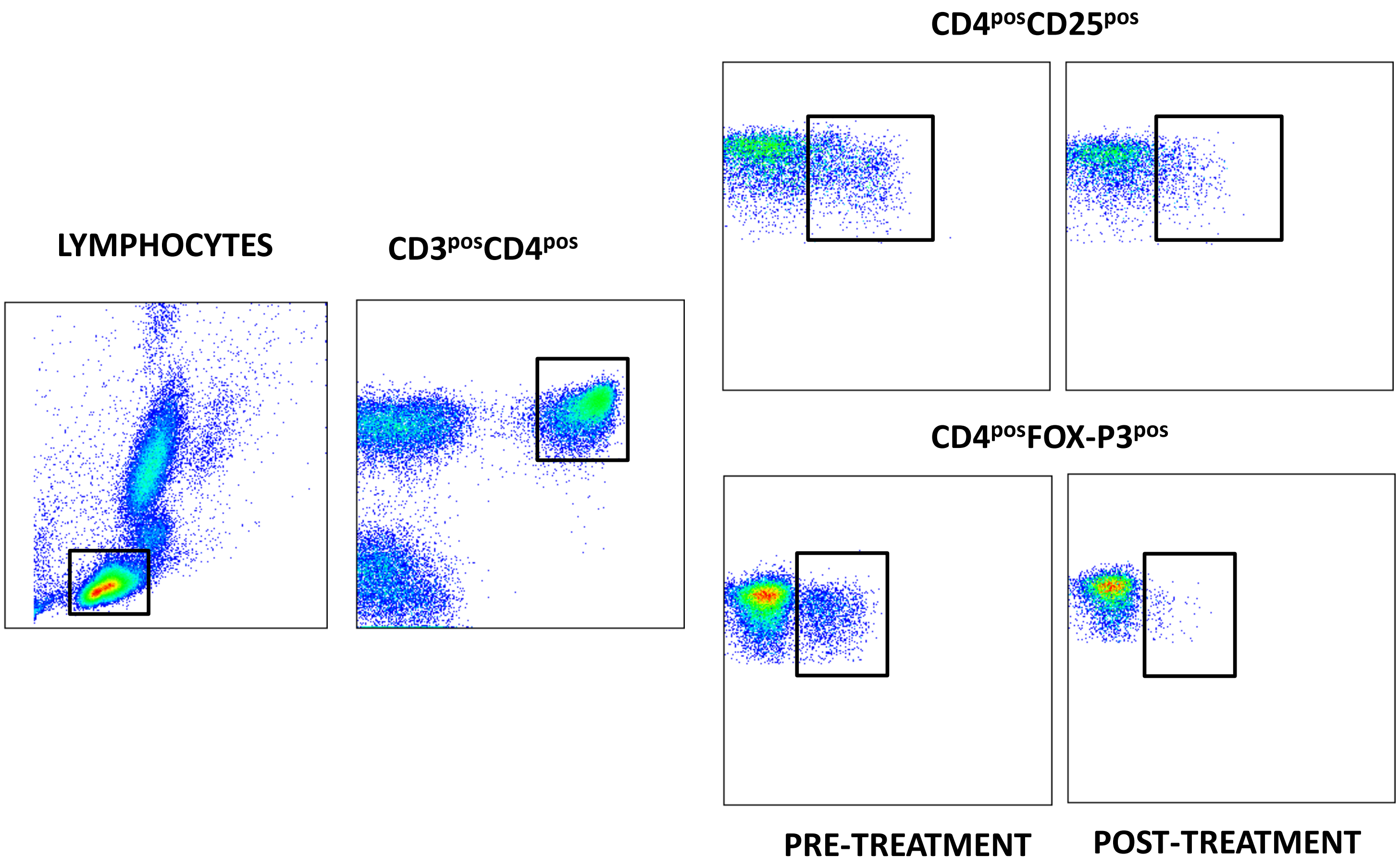

Supplemental Figure S6. Gating strategy employed to characterize CD4$^+$ T cells expressing either CD25 or FoxP3 (regulatory T cells-Tregs. CD4$^+$ and CD8$^+$ T cells were gated on singlets followed by lymphocytes and CD3$^+$; CD4$^+$ T cells expressing CD25 (upper panels) or FoxP3 (lower panels) were next selected. Expression of both CD25and FoxP3 are shown before and after Ontak administration.

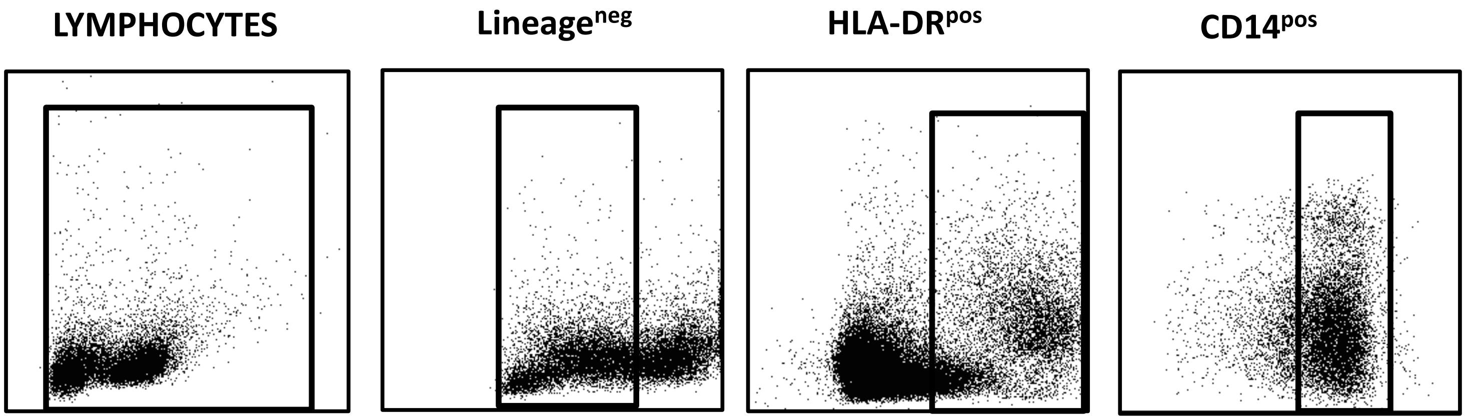

Supplemental Figure S7. Gating strategy employed to characterize monocytes/macrophages and their activation status. On the monocyte cloud, lineage negative cells were first selected, then, from this population was selected the fraction of cells expressing HLA-DR. From this population, the fraction expressing CD14 was selected.
